# Efficient N‐Type Organic Electrochemical Transistors and Field‐Effect Transistors Based on PNDI‐Copolymers Bearing Fluorinated Selenophene‐Vinylene‐Selenophenes

**DOI:** 10.1002/advs.202303837

**Published:** 2023-08-07

**Authors:** Jongho Kim, Xinglong Ren, Youcheng Zhang, Daniele Fazzi, Suraj Manikandan, Jens Wenzel Andreasen, Xiuming Sun, Sarah Ursel, Hio‐Ieng Un, Sébastien Peralta, Mingfei Xiao, James Town, Arkadios Marathianos, Stefan Roesner, Thanh‐Tuan Bui, Sabine Ludwigs, Henning Sirringhaus, Suhao Wang

**Affiliations:** ^1^ Laboratoire de Physicochimie des Polymères et des Interfaces CY Cergy Paris Université 5 Mail Gay Lussac Neuville‐sur‐Oise 95000 France; ^2^ Optoelectronics Group Cavendish Laboratory University of Cambridge JJ Thomson Avenue Cambridge CB3 0HE UK; ^3^ Dipartimento di Chimica “Giacomo Ciamician” Università di Bologna Via F. Selmi 2 Bologna 40126 Italy; ^4^ Department of Energy Conversion and Storage Technical University of Denmark Kgs. Lyngby 2800 Denmark; ^5^ IPOC‐Functional Polymers Institute of Polymer Chemistry and Center for Integrated Quantum Science and Technology(IQST) University of Stuttgart Pfaffenwaldring 55 70569 Stuttgart Germany; ^6^ Department of Chemistry University of Warwick Gibbet Hill Road Coventry CV4 7AL UK; ^7^ Present address: Department of Textile System Eng. Kyungpook National University Daegu 41566 Republic of Korea

**Keywords:** intermolecular packing, mixed ionic–electronic conductors, n‐type conjugated polymers, organic electrochemical transistors, organic field‐effect transistors

## Abstract

n‐Type organic electrochemical transistors (OECTs) and organic field‐effect transistors (OFETs) are less developed than their p‐type counterparts. Herein, polynaphthalenediimide (PNDI)‐based copolymers bearing novel fluorinated selenophene‐vinylene‐selenophene (FSVS) units as efficient materials for both n‐type OECTs and n‐type OFETs are reported. The PNDI polymers with oligo(ethylene glycol) (EG7) side chains P(NDIEG7‐FSVS), affords a high µC* of > 0.2 F cm^−1^ V^−1^ s^−1^, outperforming the benchmark n‐type Pg4NDI‐T2 and Pg4NDI‐gT2 by two orders of magnitude. The deep‐lying LUMO of −4.63 eV endows P(NDIEG7‐FSVS) with an ultra‐low threshold voltage of 0.16 V. Moreover, the conjugated polymer with octyldodecyl (OD) side chains P(NDIOD‐FSVS) exhibits a surprisingly low energetic disorder with an Urbach energy of 36 meV and an ultra‐low activation energy of 39 meV, resulting in high electron mobility of up to 0.32 cm^2^ V^−1^ s^−1^ in n‐type OFETs. These results demonstrate the great potential for simultaneously achieving a lower LUMO and a tighter intermolecular packing for the next‐generation efficient n‐type organic electronics.

## Graphical abstract

Novel PNDI‐based polymers bearing fluorinated selenophene‐vinylene‐selenophene (FSVS) are synthesized. The glycolated P(NDIEG7‐FSVS) with a deep‐lying LUMO of −4.63 eV gives an ultra‐low threshold voltage of 0.16 V, yielding a µC* > 0.2 F cm^−1^ V^−1^ s^−1^ in n‐type OECTs; the alkylated P(NDIOD‐FSVS), with a low Urbach energy of 36 meV and a low activation energy of 39 meV, exhibits electron mobility of > 0.3 cm^2^ V^−1^ s^−1^ in n‐type OFETs.

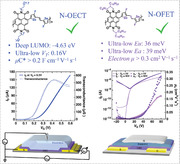

## 1 Introduction

Organic mixed ionic‐electronic conductors (OMIECs) can transfer and couple ionic and electronic charges^[^ ^1^ ^]^ making them a viable foundation for various emerging organic electronic and electrochemical devices including organic thermoelectric (OTE) devices^[^ ^2^ ^]^ and organic electrochemical transistors (OECTs).^[^ ^1^, ^3^ ^]^ Particularly, OECTs are 3‐terminal devices in which the electrical conductivity of an organic semiconductor is modulated by the gate voltage, which controls the injection of ions and charge carriers into the bulk of the semiconductor layer.^[^ ^4^ ^]^ This is in contrast to conventional organic field‐effect transistors (OFETs) where charge transport mainly occurs near the interface of the organic semiconductor and the dielectric layer.^[^ ^5^ ^]^ OECTs can be operated at low voltages because of their electrolyte‐gated nature, and volumetric charging enables them to transduce and amplify subtle voltage signals, resulting in higher sensitivity compared to that of conventional OFETs.^[^ ^6^ ^]^ OECTs have also been extensively explored for use in bioelectronics, where they can interface with biological systems such as cells and tissues.^[^ ^7^ ^]^ Overall, the combination of biocompatibility, low‐voltage operation, and large‐signal amplification, makes OECTs attractive for a wide range of applications, including biosensors, flexible electronics, and implantable medical devices.

As a performance metric, the performance figure‐of‐merit for OECTs, *µ*C* (the product of the mobility and volumetric capacitance),^[^ ^8^ ^]^ has been greatly improved over the last decade. Thus far, p‐type polymers have been extensively developed. For instance, the most widely investigated p‐type system, PEDOT:PSS has a figure‐of‐merit *µ*C* of ≈50 F cm^−1^ V^‐−1^ s^−1^.^[^ ^7b^ ^]^ Remarkably, *µ*C* > 500 F cm^−1^ V^−1^ s^−1^ was recently reported for the state‐of‐the‐art polythiophene‐based OECTs.^[^ ^9^ ^]^ However, the development of n‐type OECTs is still lagging, mainly because of the generally lower *µ*C* caused by the low electron mobility (*µ*_el_) of bulk doped polymers, as well as the instability of n‐type polymers under ambient conditions.^[^ ^10^ ^]^ To date, only a few types of polymers are known to exhibit µC* figure‐of‐merit of over 1 F cm^−1^ V^−1^ s^–1^.^[^ ^11^ ^]^ The first polymer used for stable n‐type OECTs, Pg4NDI‐T2 was reported by McCulloch et al. in 2016.^[^ ^12^ ^]^ In that pioneering study, to improve ion penetration in the polymer, hydrophilic glycol side chains were employed to replace the octyldodecyl side chains in the benchmark n‐type P(NDI2OD‐T2), which has been extensively investigated as OFET materials.^[^ ^13^ ^]^ Despite its encouraging stability, the performance of Pg4NDI‐T2 in an OECT was far from satisfactory, with a *µ*_el_ of only 7.3×10^−6^ cm^2^ V^−1^ s^−1^ and a low µC* of 1.6×10^−3^ F cm^−1^ V^−1^ s^−1^.^[^ ^12^ ^]^ Since then, naphthaplenediimide (NDI) has been recognized as a benchmark n‐type building block, and significant efforts have been made to improve its device performance, making PNDI‐based derivatives the most investigated group of materials for n‐type OECTs. Notably, to improve the performance of OECTs, the volumetric capacitance needs to be maximized while maintaining a sufficiently high electron mobility after electrochemical doping. To date, side chain engineering is the most commonly used molecular design strategy for tuning the performance of NDI‐based OECTs.^[^ ^10b^ ^]^ For example, the working mode of PNDI‐T2‐based copolymers can be controlled by changing the ethylene glycol/alkyl side chains ratio.^[^ ^14^ ^]^ However, there is a tradeoff in which increasing the amount of ethylene glycol side chains significantly increases the capacitance, accompanied by an undesirable decline in the *µ*_el_ by over two orders of magnitude to the level of 10^−4^ cm^2^ V^−1^ s^−1^ due to the disruption of the intermolecular aggregations caused by the ethylene glycol side chains. The highest performance was achieved with P‐90 (90% of glycol side chains), rather than with P‐100.^[^ ^14^ ^]^ Other side‐chain engineering strategies reported to date include the introduction of alkyl spacers into linear glycol side chains^[^ ^15^ ^]^ and the use of branched side chains to minimize the disruption of structural ordering and charge delocalization.^[^ ^16^ ^]^ In addition to PNDI‐based derivatives, there are a few other classes of polymers that have been demonstrated to be promising in n‐type OECTs, including the ladder‐type polymer poly(benzimidazobenzophenanthroline) (BBL),^[^ ^17^ ^]^ fused lactone polymers,^[^ ^18^ ^]^ diketopyrrolopyrrole derivatives^[^ ^19^ ^]^ as well as bithiophene Imide derivatives.^[^ ^20^ ^]^ Generally, the field of OECTs is limited mainly by the scarcity of n‐type polymers with high OECT performance. In addition, the understanding of the structure‐property relationships remains fragmented. Because building organic complementary circuits and multi‐sensor arrays necessitates both p‐type and n‐type OECTs, it is thus of vital importance to develop efficient n‐type polymers for higher performance and to achieve a better understanding of their structure‐performance relationship. Heteroatom (e.g., F and Se) substitution has been effectively employed in tuning the electronic structures of conjugated polymers in OFETs and OTEs; however, this approach has rarely been reported for OECTs.

**Figure 1:**
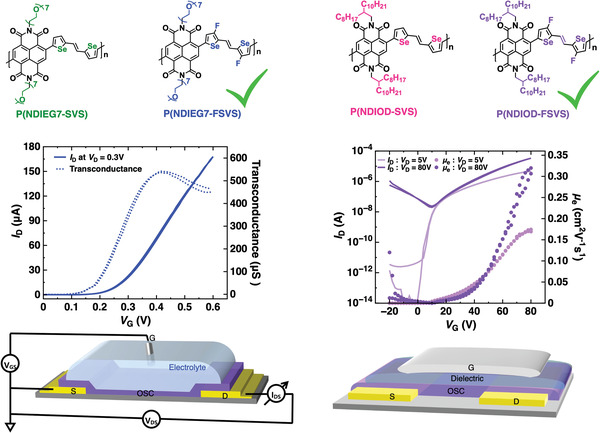
Molecular structures of the polymers; OECT and OFET transfer curves for the fluorinated polymers and schematic illustration of the devices.

Herein, we report novel PNDI‐based polymers bearing both F and Se (fluorinated selenophene‐vinylene‐selenophene (FSVS)) on the donor part of the backbone for achieving efficient OECTs and OFETs. (**Figure** **1**). The PNDI‐based polymers bearing SVS are used as the reference polymers. P(NDIEG7‐SVS) exhibits a deep LUMO of −4.39 eV, whereas fluorination of the P(NDIEG7‐SVS) leads to a further decrease in the LUMO by −0.24 eV down to as low as −4.63 eV, as supported by Density Functional Theory (DFT) calculations. As a result, the threshold voltage is reduced from 0.37 V for P(NDIEG7‐SVS) to 0.16 V for P(NDIEG7‐FSVS), which is an ultra‐low threshold value for n‐type OECTs. Moreover, P(NDIOD‐FSVS) exhibits surprisingly low energetic disorder, with an Urbach energy of 36 meV and an ultra‐low activation energy of 39 meV, leading to a high electron mobility of up to 0.32 cm^2^ V^−1^ s^−1^ in n‐type OFETs. Compared to the relevant reference P(NDIOD‐SVS) which predominately adopts face‐on orientation in solid‐state thin films, P(NDIOD‐FSVS) exhibits primarily edge‐on orientation and a shorter π‐π stacking distance, as indicated by X‐ray diffraction measurements.

## 2 Results and Discussion

**Scheme 1:**
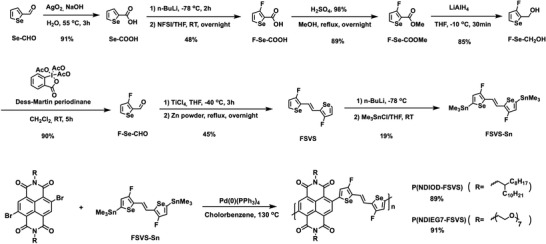
Synthetic routes to the FSVS‐Sn and its corresponding polymers P(NDIOD‐FSVS) and P(NDIEG7‐FSVS).

The routes for synthesizing the novel donor FSVS and the corresponding polymers are shown in **Scheme** **1**; the details are presented in the Supporting Information. Starting from commercially available **Se‐CHO**, the aldehyde functionality was oxidized followed by selective fluorination to produce **FSe‐COOH**. After forming the methyl ester, **FSe‐COOMe** was reduced to the primary alcohol, **FSe‐CH_2_OH**. Careful control of the reaction conditions was required to avoid product defluorination. DMP oxidation followed by McMurry coupling provided **FSVS**, which was further converted to the selenophene building block, **FSVS‐Sn**. Although treatment of **FSVS** with *n*BuLi caused significant defluorination, the use of LDA prevented this side‐reaction.

**Figure 2:**
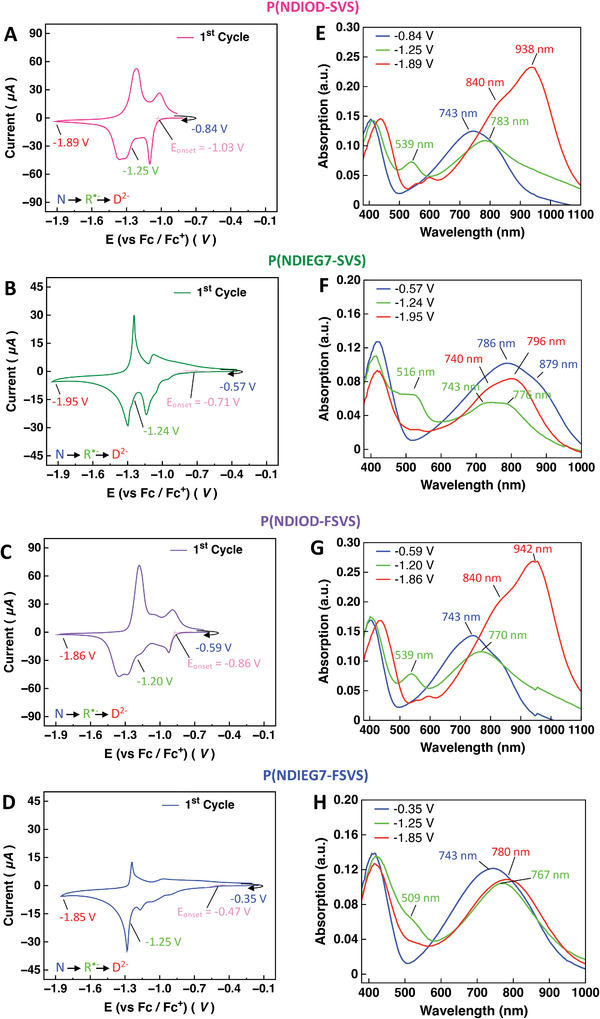
In situ spectroelectrochemical data for polymer films. Cyclic voltammograms of A) P(NDIOD‐SVS) (LUMO: −4.07 V), B) P(NDIEG7‐SVS) (LUMO: −4.39 V), C) P(NDIOD‐FSVS) (LUMO: −4.24 V), and D) P(NDIEG7‐FSVS) (LUMO: −4.63 V) and characteristic absorption spectra of different redox species at indicated electrochemical potentials during reduction in the forward scan of the first cycle in E) to H).

To evaluate the electrochemical properties of PNDI‐based polymer films, cyclic voltammetry (CV) was performed during reduction, with the LUMO levels estimated from the reduction onsets for the different polymers summarized in **Table** **1**. The CV curves in **Figure** **2**A–D clearly show that the onset of reduction (n‐doping) for the OD polymers is more negative than that of EG7 counterparts, similar to observations for previously reported P(NDI‐T2) polymers.^[^ ^12^, ^21^ ^]^ Moreover, the fluorination of the SVS unit can effectively shift the LUMO levels down by 0.17–0.24 eV. As a result, a low LUMO value of –4.63 eV was recorded for P(NDIEG7‐FSVS), which, to the best of our knowledge, is the lowest‐lying LUMO energy ever reported for NDI‐based polymers. Notably, polymers with LUMO levels deeper than −4.0 eV can meet the energy level requirement for stable n‐type OECT operation under ambient conditions.^[^ ^12^, ^22^ ^]^

**Table 1:** Summary of polymer properties

Polymer	Mn [kg mol^−1^]	D	Eg [eV] ^a)^	E_LUMO_ [eV]^b)^	E_LUMO_ [eV]^c)^
P(NDIOD‐SVS)	17.9	2.47	1.32	−4.07	−4.05
P(NDIEG7‐SVS)	10.1	2.94	1.18	−4.39	−4.41
P(NDIOD‐FSVS)	11.0	2.29	1.28	−4.24	−4.22
P(NDIEG7‐FSVS)	6.6	2.52	1.28	−4.63	−4.62

^a)^ Optical bandgap from UV–vis.

^b)^ LUMO from CV reduction onset.

^c)^ LUMO from spectral onsets during reduction measured with in situ spectroelectrochemistry.

Since onset determination (and therefore LUMO determination) using CV measurements is often rather unreliable and onset determination is particularly difficult for P(NDIEG7‐SVS) and P(NDIEG7‐FSVS), we also applied spectral onset determination using in‐situ spectroelectrochemistry measurements.^[^ ^23^ ^]^ This method was established to characterize homopolymers and blends for organic solar cell energy level characterization and also proved to give reliable data for conjugated polyelectrolytes.^[^ ^22^ ^]^ Here, we performed a spectral onset determination from the evolution of the absorption intensity of the neutral and first reduced state during reduction for all four polymers. (Figure S3, Table S1, Supporting Information) The corresponding characteristic absorption spectra are shown in Figure 2E–H and Figure S9 (Supporting Information). The spectra were recorded at the indicated reduction potentials during the forward scan of the first cycle. According to the previous investigation of P(NDI2OD‐T2), it is well‐known that reduction from the neutral state with absorption maxima at 389 and 674 nm leads to a first reduced state with a maximum at 489 nm and a second reduced state with characteristic bands at 395 and 718 nm.^[^ ^21a^ ^]^ The first reduced state is assigned as a radical anion species and the second reduced state is a dianion species. The UV–vis spectrum of the neutral state of P(NDIOD‐SVS) showed two main absorption bands at 405 and 743 nm (Figure 2E, blue). In the potential range of −0.84 to −1.25 V, the band at 405 nm shifted toward 412 nm and the band at 743 nm continuously decreases, while two new bands at 539 and 783 nm appeared which were assigned to the radical anion (Figure 2E, green). The bands at 743 nm (neutral) and 539 nm (radical anion) were used to determine the LUMO (Figure S3, Supporting Information). During reduction from −1.25 to −1.89 V, the band at 412 nm shifted to 438 nm and the intensity of absorption band of the radical anion at 539 nm decreased, while new bands appeared at 599 and 938 nm, with a shoulder ≈840 nm (Figure 2E, red). The absorption spectra of the other three polymers were quite similar (see Figure 2F–H with the indicated absorption maxima). The radical anion intermediate state could be easily identified owing to its characteristic maximum of around 500 nm. Interestingly, for the polymers with the EG‐side group, the absorption of the neutral and the dianion species strongly overlapped compared to that of the OD‐polymers. To gain a deeper understanding of this phenomenon, in‐depth measurements will be performed in future studies.

**Figure 3:**
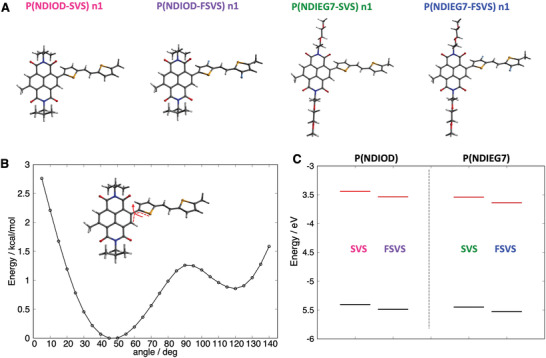
A) DFT (B3LYP/ZORA‐def2‐ZZVP) optimized molecular structures for the monomer (n1) of P(NDIOD‐SVS), P(NDIOD‐FSVS), P(NDIEG7‐SVS), and P(NDIEG7‐FSVS). B) DFT torsional energy profile computed for the monomers of P(NDIOD‐SVS) species (similar results were obtained for the other species). C) Computed frontier molecular orbital energies (HOMO, black, and LUMO, red) for the monomers of P(NDIOD) and P(NDIEG7) without and with fluorine substitution.

To gain further insights into the structural and electronic properties of all the polymers, tight‐binding semiempirical (GFN2‐xTB) and DFT calculations were performed (see Computational Methods). An oligomer approach was adopted, considering both a monomer unit (called n1) and a tetramer, that is, an oligomer featuring four repeat units (n4). **Figure** **3**A shows the DFT‐optimized structures of the most stable conformers of the P(NDIOD‐SVS), P(NDIOD‐FSVS), P(NDIEG7‐SVS), and P(NDIEG7‐FSVS) monomers. The structures are very similar, with a dihedral angle of ca. 40–43° between the selenophene ring and the NDI plane, whereas the SVS and FSVS moieties are completely flat owing to the vinylene bridge. Figure 3B shows a prototypical torsional energy profile for both the SVS‐ and FSVS‐based polymers. The computed potential curve exhibits a double‐minima profile, with the most stable conformer at ≈45°, and the less stable conformer, ≈1 kcal mol^−1^ higher in energy, at 120°. For longer oligomers, namely, the tetramer n4, the computed intramolecular structures preserve the dihedral angle between the selenophene and the NDI units for each polymer (see Supporting Information). Figure 3C shows the computed DFT orbital energies for the highest and lowest occupied molecular orbitals (HOMO and LUMO, respectively) of the monomer species (similar results hold for the tetramer n4, see Supporting Information). The fluorinated species (PNDI–FSVS) had lower LUMO energies than the non‐fluorinated polymers. This stabilization is related to the presence of the fluorine atoms, which partially delocalize the orbital on the donor SVS unit. Although the absolute orbital energies cannot be directly compared with the experimental values (owing to the well‐known limitations of DFT and the choice of the exchange‐correlation function), the relative energy variations can be instead correlated. The decrease in the LUMO energy upon fluorination (FSVS) was in very good agreement with the experimental data. Furthermore, the computed LUMO energies for the oligomers of PNDIEG7 species were lower than those of the respective PNDIOD species (see Supporting Information), well supporting the experimental data.

**Figure 4:**
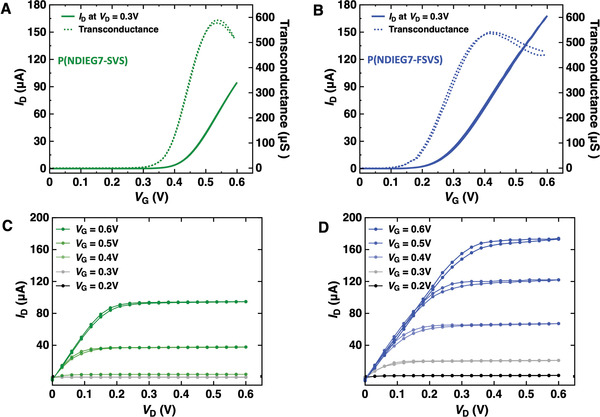
Transfer curves for organic electrochemical transistors employing A) P(NDIEG7‐SVS) (µC* = 0.21±0.01 F cm^−1^ V^−1^ s^−1^; g_m_, _norm_ = 0.041±0.01; VT = 0.35±0.01; n = 3), and B) P(NDIEG7‐FSVS), (µC* = 0.25±0.07 F cm^−1^ V^−1^ s^−1^; g_m_, _norm_ = 0.046±0.001; VT = 0.16±0.01; n = 3); output curves for C) P(NDIEG7‐SVS), and D) P(NDIEG7‐FSVS). (n = 3).

The transfer and output characteristics of the OECTs employing P(NDIEG7‐SVS) and P(NDIEG7‐FSVS) are presented in **Figure** **4**. Both devices exhibit typical n‐type characteristics with negligible hysteresis, indicating fast and efficient ion injection. The devices employing P(NDIOD‐SVS) and P(NDIOD‐FSVS) did not exhibit any detectable signals (*I_D_* was lower than *I_G_*). These results indicate that the presence of the EG side chain plays a critical role in facilitating the diffusion of ions through the bulk of the polymer layer, thereby improving the OECT performance by several orders of magnitude. The threshold voltage of the device with P(NDIEG7‐FSVS) was smaller than that of the congener with P(NDIEG7‐SVS) (0.16 V vs 0.35 V), consistent with the deeper LUMO of the former. Notably, the threshold voltage of P(NDIEG7‐FSVS) is one of the lowest values ever reported for n‐type OECTs. Despite their different threshold voltages, the geometry‐normalized transconductance (≈0.04 S cm^−1^) and OECT figures‐of‐merit (µC* ≈ 0.21–0.25 F cm^−1^ V^−1^ s^−1^) for the OECTs employing the two polymers were similar. Note that the figures‐of‐merit for P(NDIEG7‐SVS) and P(NDIEG7‐FSVS) are in the same order as that of the ladder‐type polymer BBL^[^ ^17^ ^]^ and the fused lactone polymers PgNaN.^[^ ^18^ ^]^ As shown in Figure S9 (Supporting Information), the capacitance of P(NDIEG7‐FSVS) is quite similar to that of the previously reported PNDI‐based P‐90.^[^ ^14^ ^]^ Thus, it is apparent that the significantly higher µC* of P(NDIEG7‐SVS) and P(NDIEG7‐FSVS) predominantly stems from the increase in the mobility of the OECT. Note that the present values were obtained from polymer films without any optimization of the processing conditions, purity levels, or molecular weights, which were found to significantly affect the device performance of both n‐type and p‐type OECT devices.^[^ ^24^ ^]^ Therefore, it is anticipated that the performance of the developed NDI‐based OECTs can be further improved by optimization.

**Figure 5:**
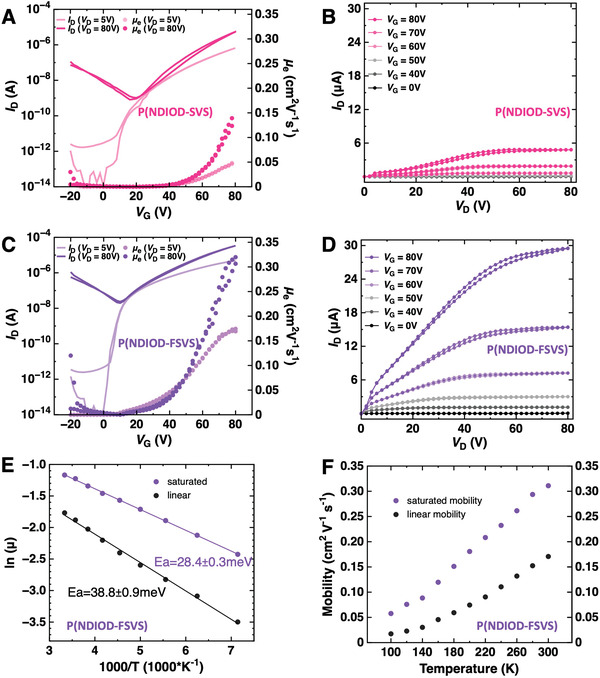
FET characteristics for OFET devices with a top‐gate, bottom‐contact geometry of P(NDIOD‐SVS) A) transfer, B) output characteristics showing a µe of 0.15 cm^2^ V^−1^ s^−1^ (0.14±0.01 cm^2^ V^−1^ s^−1^, n = 3) C) Transfer and D) output characteristics for P(NDIOD‐FSVS) showing a µe of 0.32 cm^2^ V^−1^ s^−1^ (0.30±0.02 cm^2^ V^−1^ s^−1^, n = 3) E) Arrhenius plots and F) temperature‐dependent linear and saturation mobility values extracted at VG = 80 V for P(NDIOD‐FSVS) FET devices.

We further evaluated the electrical performance and charge transport properties of top‐gate, bottom‐contact FETs. The transfer and output characteristics of the OFETs with P(NDIOD‐SVS) and P(NDIOD‐FSVS) are shown in **Figure** **5**A,B,C,D, respectively. All the devices displayed typical n‐type transport characteristics. Significantly, the PNDIOD‐based devices exhibited better operational stability, whereas an irreversible quasi‐self‐doping effect was generally observed for the PNDIEG7‐based devices during the transfer characteristic analysis. As shown in Figure 5A, the P(NDIOD‐SVS) device exhibited an I_ON_ of 6.7 µA in the saturation regime and 0.81 µA in the linear regime, with a gate voltage‐dependent mobility (*µ_e_*) reaching values of 0.15 cm^2^ V^−1^ s^−1^ in the saturation regime and 0.05 cm^2^ V^−1^ s^−1^ in the linear regime, and a current modulation of over 10^5^. Remarkably, the fluorination of the SVS group effectively improved the electrical performance of the OFET devices. As shown in Figure 5B, P(NDIOD‐FSVS) exhibited a much higher I_ON_ of 34 µA in the saturation regime and 4.8 µA in the linear regime, with a *µ_e_* of 0.32 cm^2^ V^−1^ s^−1^ in the saturation regime and 0.17 cm^2^ V^−1^ s^−1^ in the linear regime, and a current modulation of over 10^6^. Such a high electron mobility is very comparable to that of the recently reported high‐performance n‐type ultra‐rigid copolymer BNBP‐BBTz (0.34 cm^2^ V^−1^ s^−1^),^[^ ^25^ ^]^ and efficient n‐type fused lactone polymers AN (0.23 cm^2^ V^−1^ s^−1^) and NN (0.33 cm^2^ V^−1^ s^−1^).^[^ ^26^ ^]^ As shown in Figure 5C,D, the output curves of P(NDIOD‐SVS) and P(NDIOD‐FSVS) both exhibited a well‐defined saturation regime with low injection barriers. Note that the origin of the non‐ideal kink in the low *V_D_* region of the Figure 5D may be due to non‐uniformities of the polymer films leading to early saturation of the current in parts of the channel(Figure S10, Supporting Information). In the OFETs of both polymers, the gate‐voltage dependence of the mobility suggests that further device optimization may be achieved through future optimization of the contact resistance and film morphology.

High‐performance P(NDIOD‐FSVS) was subjected to temperature‐dependent transfer characteristics measurements in the range of 140–300 K (Figure 5E; Figure S11, Supporting Information). P(NDIOD‐FSVS) exhibits typical temperature‐activated electron transport, as evidenced by the linear increase in the electron mobility with increasing temperature, as shown in Figure 5F and the corresponding Arrhenius plots in Figure 5E. The activation energy (*E_a_*) was 28.4 ±0.3 meV in the saturation regime (*V_D_* = 80 V) and 39 ± 1 meV in the linear regime (V_D_ = 5 V). The negative dependence of E_a_ on the drain voltage has been reported previously.^[^ ^27^ ^]^ Note that these activation energy values for P(NDIOD‐FSVS) are lower than those for previously reported high‐performance conjugated polymers, such as the classic semi‐crystalline polymer PBTTT(55–58 meV),^[^ ^28^ ^]^ the benchmark n‐type P(NDI2OD‐T2) (44 meV with CYTOP and 64 meV with polystyrene as dielectric layers),^[^ ^13b^ ^]^ the disorder‐free polymer IDTBT (61 meV)^[^ ^29^ ^]^ as well as the double‐stranded rigid‐rod fused‐ring polymer NN (106 meV).^[^ ^30^ ^]^ The low activation energy indicates an energetic landscape for charge transport with a low degree of energetic disorder and also reflects the use of CYTOP with low a dielectric constant of 2.1 which is effective in inducing less dipolar disorder compared to PMMA and SiO_2_.^[^ ^13b^ ^]^

**Figure 6:**
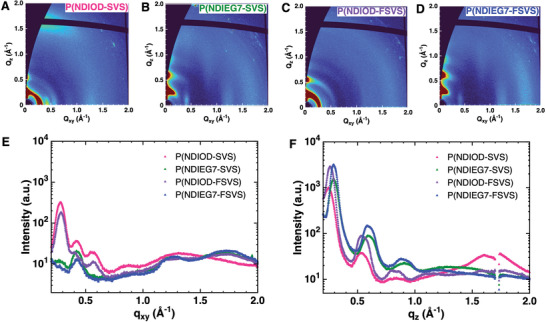
2D GIWAXS patterns of A) P(NDIOD‐SVS), B) P(NDIEG7‐SVS), C) P(NDIOD‐FSVS), and D) P(NDIEG7‐FSVS). Linecuts in the E) in‐plane and F) out‐of‐plane directions for all four polymers.

2D Grazing‐incidence wide‐angle X‐ray scattering (GIWAXS) was used to elucidate the solid‐state microstructures of the polymers; the corresponding results are shown in **Figure** **6** and **Table** **2**. As shown in the 2D diffraction patterns in Figure 6A, P(NDIOD‐SVS) adopted a face‐on orientation, as indicated by the strong out‐of‐plane strong (0k0) peak with a *q*‐spacing of 1.67 Å^−1^ (also shown in Figure 6F), corresponding to a π‐π staking distance of 3.8 Å, and the lamellar stacking reflection at a *q*‐spacing of 0.24 Å^−1^ along both the in‐plane and out‐of‐plane directions. In contrast, P(NDIOD‐FSVS) exhibits edge‐on orientation, as suggested by the weaker in‐plane (010) peak with a q‐spacing of 1.69 Å^−1^ (also shown in Figure 6E), corresponding to a shorter π‐π staking distance of 3.7 Å as well as lamellar packing as indicated by the out‐of‐plane peaks shown in Figure 6C. The transition from face‐on to edge‐on orientation and the shorter π‐π stacking distance are consistent with the observed larger electron mobility observed for the P(NDIOD‐FSVS) OFET devices. Previously, it has been reported that a shorter π‐π stacking distance facilitates faster charge transport.^[^ ^31^ ^]^ Additionally, the larger q‐spacing of P(NDIOD‐FSVS) corresponds to a shorter lamellar packing distance (2.51 nm) compared to that of P(NDIOD‐SVS) (2.62 nm). Hence, the introduction of F atoms led to tighter packing (both for π‐π stacking and lamellar stacking), most likely due to the Se‐F non‐covalent interactions.

**Table 2:** GIWAXS quantitative data

Polymer	Lamellar stacking			π‐π stacking		
	q [Å^−1^]	d [Å]	CL [nm] ^a)^	q [Å^−1^]	d [Å]	CL [nm] ^a)^
P(NDIOD‐SVS)	0.24	26.2	245.9	1.67	3.8	21.6
P(NDIEG7‐SVS)	0.25	25.6	245.9	1.69	3.7	16.6
P(NDIOD‐FSVS)	0.25	25.1	377.0	1.69	3.7	14.9
P(NDIEG7‐FSVS)	0.29	21.7	353.4	1.72	3.7	15.3

^a)^ CL = coherence length.

Despite the shorter packing distances in both directions, there is no evidence that the semi‐crystalline order of P(NDIOD‐FSVS) is higher than that of P(NDIOD‐SVS) as indicated by the broad arcs of the scattering at a radial of ≈0.54 and 1.65 Å^−1^ (Figure 6C). This is consistent with previous reports showing that long‐range crystallinity is not always essential for efficient charge transport.^[^ ^21^, ^32^ ^]^ In addition, the glycolated polymers show closer π‐π stacking and shorter lamellar stacking distances than their corresponding alkylated polymers with the same donor (Table 2). This is mainly due to the fact that the glycolated chains are less bulky, resulting in less steric hindrance and thus tighter intermolecular packing.

**Figure 7:**
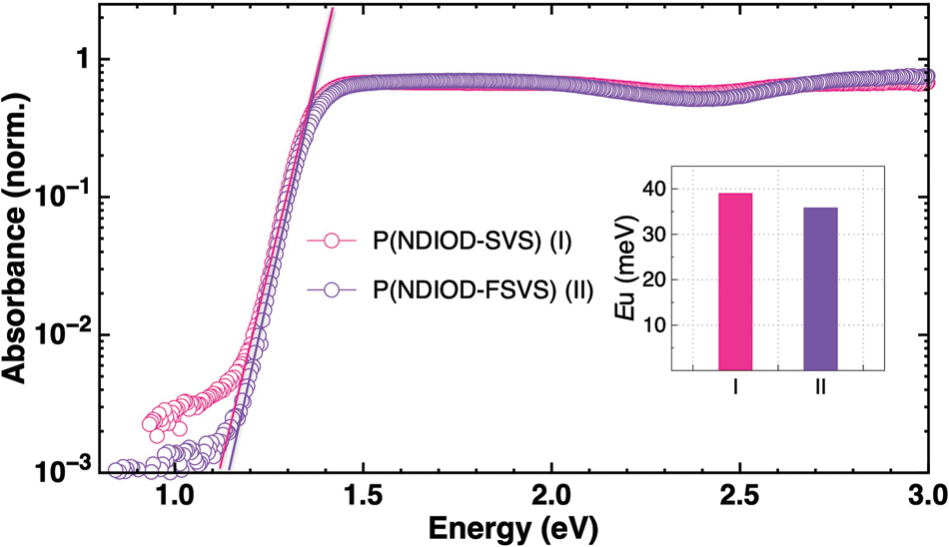
Energetic disorder probed using photothermal deflection spectroscopy. Inset figure shows the Urbach energy, EU, for both polymers.

To gain further insights into the origin of the high electron mobilities of these polymers, photothermal deflection spectroscopy (PDS) was employed to probe the energetic disorder which is manifested as sub‐bandgap tail states. By fitting the absorbance tail over the edge of the energy gap with the correlation of the absorption data (∝ exp[(E – E_g_)/ E_U_]), the Urbach energy(*E_U_*) was determined to be 39 meV for P(NDIOD‐SVS) and 36 meV for P(NDIOD‐FSVS) (**Figure** **7**). Unlike the invariant *E_U_* values observed for IDT‐, IDTT‐, and TIF‐based copolymers upon F‐substitution of the BT unit,^[^ ^33^ ^]^ the F‐substitution in the SVS donor units shown here marginally reduces *E_U_* by 3 meV. This can be rationalized by the GIWAXS data, which show that the introduction of F atoms induces stronger intermolecular aggregation and thus, a lower degree of conformational disorder. The extracted *E_U_* values are comparable to those of other high‐mobility D‐A copolymers and lower than those of the best‐performing semicrystalline polymer PBTTT.^[^ ^32a^ ^]^ It has been found that the *E_U_* is closely correlated with the energetic disorder T_0_ in the transport density of states (DOS), and a low E_U_ value is required for ideal (MOSFET‐like) transistor behaviors with a gate‐voltage independent mobility.^[^ ^34^ ^]^ For the P(NDIOD‐SVS) and P(NDIOD‐FSVS) OFETs, the mobilities extracted from the transfer characteristics are dependent on gate voltage and drain voltages. This suggests that further device optimization is required in the future to exploit the low energetic disorder that can be realized in these polymers.

## 3 Conclusion

The novel fluorinated P(NDIOD‐FSVS) and P(NDIEG7‐FSVS) were synthesized, characterized, and used as n‐type OECT and n‐type OFET materials. P(NDIEG7‐FSVS), with a deep‐lying LUMO of −4.63 eV affords an ultra‐low threshold voltage of 0.16 V, with a µC* > 0.2 F cm^‐1^ V^‐1^ s^‐1^, outperforming the benchmark n‐type Pg4NDI‐T2 and Pg4NDI‐gT2 by two orders of magnitude. Remarkably, P(NDIOD‐FSVS) exhibits an efficient charge transport behavior with an electron mobility of up to 0.32 cm^2^ V^‐1^ s^‐1^ in n‐type OFETs, with a low Urbach energy of 36 meV and a low activation energy of 39 meV measured. Both semi‐empirical tight‐binding DFT(GFN2‐xTB) and DFT calculations revealed a dihedral angle of ca. 40° between the NDI and the SVS/FSVS moieties for all the polymers, regardless of the side chains, and corroborated the experimentally observed gradual lowering of the LUMO energy for the fluorinated species. X‐ray diffraction measurement indicates that P(NDIOD‐SVS) adopts predominantly face‐on orientation in solid‐state thin films, whereas P(NDIOD‐FSVS) exhibits primarily edge‐on orientation as well as shorter π‐π stacking distances. This work highlights that lowering the LUMO and increasing the intra‐ and intermolecular interactions by fluorination and selenium substitution are effective strategies for realizing state‐of‐the‐art n‐type OECTs based on the widely studied class of PNDI copolymers, which holds great promise for guiding the design of the next‐generation efficient n‐type organic electronics.

## 4 Experimental Section

### In Situ Spectroelectrochemistry Measurements

Each of the PNDI‐based polymers was dissolved in chloroform at a concentration of 5 mg mL^−1^. All solutions were stirred at 30 °C for 30 min before spincoating on ITO (indium tin oxide) substrates. The ITO substrates had been cleaned by ultrasonication in water, isopropanol and acetone beforehand. For each film, 40 µL solution was dropped onto the substrate and spincoating conditions were 2500 rpm for 60 s followed by 3000 rpm for 30 s. All films were prepared and stored in a glovebox. All cyclic voltammograms were recorded in 0.1 M TBAPF_6_ in acetonitrile as electrolyte at a scan rate of 20 Mv s^−1^ with a three‐electrode setup using a Metrohm PGSTAT204 potentiostat. The measurements were performed at room temperature and under Argon atmosphere. The polymer film covered ITO substrates were used as working electrodes, the counter electrode was made of a Pt wire and a AgCl covered Ag wire was used as a pseudo‐reference. After the measurements, all potentials were rescaled to the ferrocene/ferrocenium (Fc/Fc^+^) couple which was used as an internal standard. For the in situ spectroelectrochemical characterization of the films, a UV–vis spectrometer equipped with an MCS621 vis II detector and a CLH600 F halogen lamp from Zeiss was utilized in parallel with the potentiostat.

The CV curves in Figures 2A–D in the main text show the reduction behavior of the four polymer films in the first voltammetric cycle, respectively. Reduction onset determination was performed using the tangent method. Table S1 (Supporting Information) summarizes the onset potentials versus Fc/Fc^+^. The LUMO values were calculated according to equation 1 ^[^ ^35^ ^]^ and were summarized in Figure 2 in the main text.

(1) $$E_{\mathrm{LUMO}}=-\left(E_{\mathrm{onset}}+5.1\right)\left[\mathrm{eV}\right]$$

Since onset potentials from CV do not always provide enough accuracy, the recently established approach was employed to use the spectral evolution of the diminishing and evolving intensity of absorption bands of the neutral and first reduced redox states, Figure S3 (Supporting Information).^[^ ^23^ ^]^ The electrochemical potential, at which the absorption intensity of the neutral species decreases and the intensity of the radical anion species increases, can be determined by the tangent method, the corresponding spectral reduction onset potential for P(NDIOD‐SVS) gives *E*_onset_ = −1.05 V. The LUMO amounts to −4.05 eV.

### GFN2‐xTB and DFT Calculations

The geometry optimization of each structure was initially performed at the semiempirical level, namely adopting the density functional tight‐binding scheme GFNn‐xTB (Geometry Frequency Non‐covalent interactions‐eXtended Tight Binding, specifically GFN2‐xTB.^[^ ^36^ ^]^ (v. 6.4.1). GFN2‐xTB meta‐dynamics were carried out with the algorithm CREST (v 2.11) for sampling the conformational space.^[^ ^37^ ^]^ Most stable conformers were optimized at the GFN2‐xTB level with very‐tight geometry‐convergence thresholds and further re‐optimized at the DFT level.

For the DFT calculations the B3LYP functional with the inclusion of dispersion correction (i.e., the atom‐pairwise dispersion correction with the Becke‐Johnson damping scheme D3BJ scheme) was considered. The basis set adopted was the def2‐TZVP (valence triple‐zeta polarization) for all atoms. To take into account relativistic effects, due to the presence of the heavy selenium atoms, the Zero‐Order Regular Approximation (ZORA) and the SARC/J auxiliary basis were included. In the structural models considered the alkyl and glycol chains have been considered however to a reduced size in order to save computational time. Tight‐binding semiempirical calculations were carried out by using the code xTB. DFT calculations were carried out by using the code ORCA v.5.0.3.^[^ ^38^ ^]^

### OECT Fabrication and Characterization

Cr/Au source/drain electrodes were fabricated on Si/SiO2 substrate using standard photolithography, evaporation, and lift‐off process. An interdigitated source/drain design (L/W = 6 um/9750 um) was chosen to get a large drain current. Polymer films were spin coated as described above, and manually patterned with a toothpick to define the channel area. OECT measurements were performed in 0.1 M aqueous solution of NaCl, with a Ag/AgCl pellet serving as the gate. The degassed electrolyte solution was confined in a PDMS well (Sylgard 184), which was prepared by mixing the base and the crosslinker (10:1 weight ratio) and baked at 60 °C for 3 h. Output and transfer characteristics were measured using an Agilent 4155B semiconductor parameter analyzer. Electrochemical impedance spectroscopy was performed on the same device architecture using a PalmSens4 potentiostat, with a frequency range between 10 000 to 1 Hz and an AC voltage amplitude of 50 mV.

### OFET Fabrication and Characterization

Top‐gate, bottom‐contact OFETs (L/W = 20 µm/1000 µm) were fabricated on glass substrates following a procedure of substrate patterning, thermal evaporation of bottom contacts (3 nm Cr and 22 nm Au), polymer spin‐coating (parameters described above), Cytop spin‐coating (2000 rpm for 20 s, annealing at 90 C for 20 mins, resulting in a thickness of 500 nm) and thermal evaporation of top gate (30 nm of Aluminum). FET characteristics were measured on a Desert TTP4 probe station by an Agilent 4155C Semiconductor Parameter Analyzer. Samples were measured under a high vacuum (<10^−5^ mbar) using liquid nitrogen as the cryogenic source.

### GIWAXS Measurement

A laboratory setup was used (Xeuss 3.0 from Xenocs S.A.). Here, supplied with a microfocus copper source, Cu Kα radiation (wavelength (λ) = 1.5418 Å) was focused and monochromatized with a 2D single reflection multilayer optic and collimated with scatterless slits. The silicon substrate surface was aligned at a grazing incident angle of 0.18° with respect to the incoming X‐ray beam. The scattered X‐ray was detected on an Eiger 4 M single‐photon counting detector, with 75 µm pixels (DECTRIS), 80.0 mm from the sample.

### PDS Measurement

Photothermal deflection spectroscopy (PDS) was performed using a tunable light source consisting of a 250 W quartz tungsten halogen lamp coupled to a 250 mm focal length grating monochromator. The monochromatic excitation beam was modulated with a mechanical chopper at 13 Hz and focused on the sample surface at a normal incidence angle. The samples were immersed in FluorinertTM FC‐72 (3 M) liquid to improve the thermooptic response in the excitation spot surroundings. Thermal gradient at the sample surface caused by nonradiative relaxation caused deflection of a probe laser beam passed parallel to the sample surface (transverse configuration), detected by a quadrant photodiode and demodulated with a lock‐in amplifier (Stanford Research Systems SR830). The sub‐gap (E_g_) absorption data A(E) were fitted to the Urbach formula assuming linear dependence of the PDS signal on the optical absorption coefficient, leading to the determination of the Urbach energy (E_U_) representing the electronic disorder: A(E) = α_0_exp[(E‐E_g_)/E_U_].

### Statistical Analysis

Both OECT and OFET measurements were repeated on three samples and then statistically analyzed to get mean values and SD. The data were presented as mean ± standard deviation (mean ± SD). Origin was used for statistical analysis.

## Conflict of Interest

The authors declare no conflict of interest.

## Author Contributions

S.W. conceived, designed, and conceptualized the research. J.K., X.R., and Y.Z. contributed equally to this work. J.K. synthesized the polymers; X.R. fabricated and measured the OECTs; Y.Z. fabricated and measured the OFETs; D.F. performed GFN2‐xTB and DFT calculations; S.M., J.W.A., and S.W. measured and analyzed GIWAXS; X.S. and S.L. performed in situ spectroelectrochemistry measurements and analyzed the results; S.U. performed the initial test of the OFETs; H.I.U. measured and analyzed PDS; S.P. performed AFM measurements; M.X. contributed to the fabrication of the electrodes for OECTs; J.T. recorded the TGA and DSC; A.M. recorded the SEC; S.R. and J.K. analyzed the NRM;S.W. organized the manuscript with contribution from all authors; D.F., S.L., H.S., and S.W. revised the manuscript; H.S. and S.W. supervised the project.

## Supporting information

Supporting Information

## Data Availability

The data that support the findings of this study are available from the corresponding author upon reasonable request.
