# Supplementary material for: Efficient N‐Type Organic Electrochemical Transistors and Field‐Effect Transistors Based on PNDI‐Copolymers Bearing Fluorinated Selenophene‐Vinylene‐Selenophenes
Source: Adv Sci (Weinh). 2023 Aug 7;10(29):2303837. doi: 10.1002/advs.202303837 (PMC10582458; doi:10.1002/advs.202303837)
Supplement: Supplementary file 1 — Supporting Information [file ADVS-10-2303837-s001.pdf]

ADVANCED SCIENCE
Open Access

Supporting Information

for *Adv. Sci.*, DOI 10.1002/advs.202303837

Efficient N-Type Organic Electrochemical Transistors and Field-Effect Transistors Based on PNDI-Copolymers Bearing Fluorinated Selenophene-Vinylene-Selenophenes

Jongho Kim, Xinglong Ren, Youcheng Zhang, Daniele Fazzi, Suraj Manikandan, Jens Wenzel Andreasen, Xiuming Sun, Sarah Ursel, Hio-Ieng Un, Sébastien Peralta, Mingfei Xiao, James Town, Arkadios Marathianos, Stefan Roesner, Thanh-Tuan Bui, Sabine Ludwigs, Henning Sirringhaus and Suhao Wang**

Supporting Information

Efficient n-type Organic Electrochemical Transistors and Field-effect Transistors based on PNDI-Copolymers Bearing Fluorinated Selenophene-Vinylene-Selenophenes

Jongho Kim, Xinglong Ren, Youcheng Zhang, Daniele Fazzi, Suraj Manikandan, Jens Wenzel Andreasen, Xiuming Sun, Sarah Ursel, Hio-Ieng Un, Sébastien Peralta, Mingfei Xiao, James Town, Arkadios Marathianos, Stefan Roesner, Thanh-Tuan Bui, Sabine Ludwigs, Henning Sirringhaus, and Suhao Wang**

J. Kim, S. Peratla, T. -T Bui, S. Wang

Laboratoire de Physicochimie des Polymères et des Interfaces, CY Cergy Paris Université, 5 Mail Gay Lussac, 95000 Neuville-sur-Oise, France

E-mail: suhao.wang1@cyu.fr

X. Ren, Y. Zhang, S. Ursel, H. -I. Un, M. Xiao, H. Sirringhaus

Optoelectronics Group, Cavendish Laboratory, University of Cambridge, JJ Thomson Avenue, Cambridge CB3 0HE, UK

E-mail: hs220@cam.ac.uk

D. Fazzi

Dipartimento di Chimica “Giacomo Ciamician”, Università di Bologna, Via F. Selmi, 2, 40126 Bologna, Italy

S. Manikandan, J. W. Andreasen

Department of Energy Conversion and Storage, Technical University of Denmark, 2800 Kgs. Lyngby, Denmark

X. Sun, S. Ludwigs

IPOC-Functional Polymers, Institute of Polymer Chemistry and Center for Integrated Quantum Science and Technology(IQST), University of Stuttgart, Pfaffenwaldring 55, 70569 Stuttgart, Germany

J. Town, A. Marathianos, S. Roesner

Department of Chemistry, University of Warwick, Gibbet Hill Road, Coventry CV4 7AL, UK

Table of Contents

1. Materials and Instrumentation, monomer and polymer synthesis, NMR Spectra
2. Polymer molecular weights, PDIs, and thermal properties
3. In-situ spectroelectrochemistry measurement
4. GFN2-xTB and DFT calculations
5. Impedance measurements for P(NDIEG7-FSVS)
6. Low-temperature transfer characteristics of P(NDIOD-FSVS) OFETs
7. AFM images of P(NDIOD-SVS) and P(NDIOD-FSVS)

1. Materials and Instrumentation, monomer and polymer synthesis, NMR Spectra

1.1. Materials and Instrumentation

4,9-Dibromo-2,7-bis(2-octyldodecyl)benzo[lmn][3,8]phenanthroline-1,3,6,8(2*H*,7*H*)-tetraone (NDIOD-Br_2) was purchased from Sunatech Inc. 2,6-Dibromonaphthalene-1,4,5,8-tetracarboxylic dianhydride (NDA-Br_2), anhydrous tetrahydrofuran (THF), titanium(IV) chloride ($TiCl_4$) (1 M in dichloromethane), H_2SO_4 (>99.8%), acetic acid (>99.5%), 2-formylselenophene (stabilized with hydroquinone) (Se-CHO), Dess-Martin periodinane (DMP), and zinc (Zn) powder were purchased from TCI. Methanol (MeOH), acetone, chloroform, and dichloromethane (CH_2Cl_2) were purchased from VWR. Tetrakis(triphenylphosphine)palladium(0) (Pd(0)(PPh_3)$_4$), and anhydrous chlorobenzene, 2-(tributylstannyl)thiophene, 2,5,8,11,14,17,20-heptaoxadocosan-22-amine (EG7-NH_2), 2.5 M *n*-butyllithium (*n*-BuLi) in THF, 1.0 M lithum aluminum hydride ($LiAlH_4$) in THF, 2.0 M lithium diisopropylamide solution (LDA) in THF/heptane/ethylbenzene and 1 M trimethyltin chloride (in THF) were purchased from Sigma Aldrich. ^{1}H and ^{19}F NMR spectra were obtained on a Bruker AvanceCore or Bruker HD400 Fourier transform spectrometer with TMS as the internal standard.

1.2. Synthesis of monomers

1.2.1. SVS

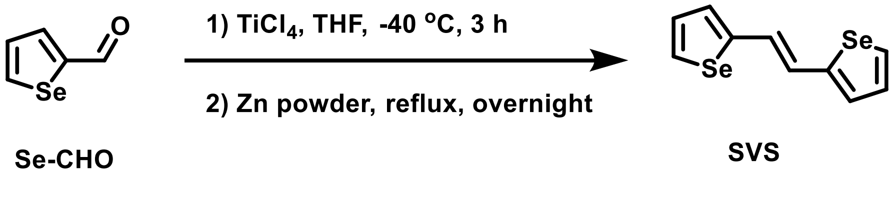

A 1 M solution of $TiCl_4$ in CH_2Cl_2 (9.43 mL, 9.43 mmol) was added drop-wise to a suspension of **Se-CHO** (1.00 g, 6.28 mmol) in THF (30 mL) at -40 °C under N_2 atmosphere, and the mixture was stirred at this temperature for 3 h. Zn powder (1.10 g, 16.8 mmol) was added portion-wise over a period for 30 min at -40 °C. The mixture was stirred at this temperature for

30 min, and then was heated under reflux overnight. The reaction was quenched by addition of cooled H_2O (50 mL). The precipitated solid was isolated by filtration and dried. The solid was dissolved in CH_2Cl_2 (50 mL) and insoluble solids were removed by filtration. The solvent was removed under reduced pressure and the residue was purified using column chromatography over neutral aluminum oxide with *n*-hexane/ CH_2Cl_2 (from 20:1 to 5:1 elution gradient). **SVS** was obtained as a yellow solid (0.56 g, 63%). ^{1}H NMR (400 MHz, $CDCl_3$): δ 7.83 (dd, J = 5.4 Hz, J = 1.4 Hz, 2H), 7.22–7.17 (m, 4H), 6.99 (s, 2H) ppm.

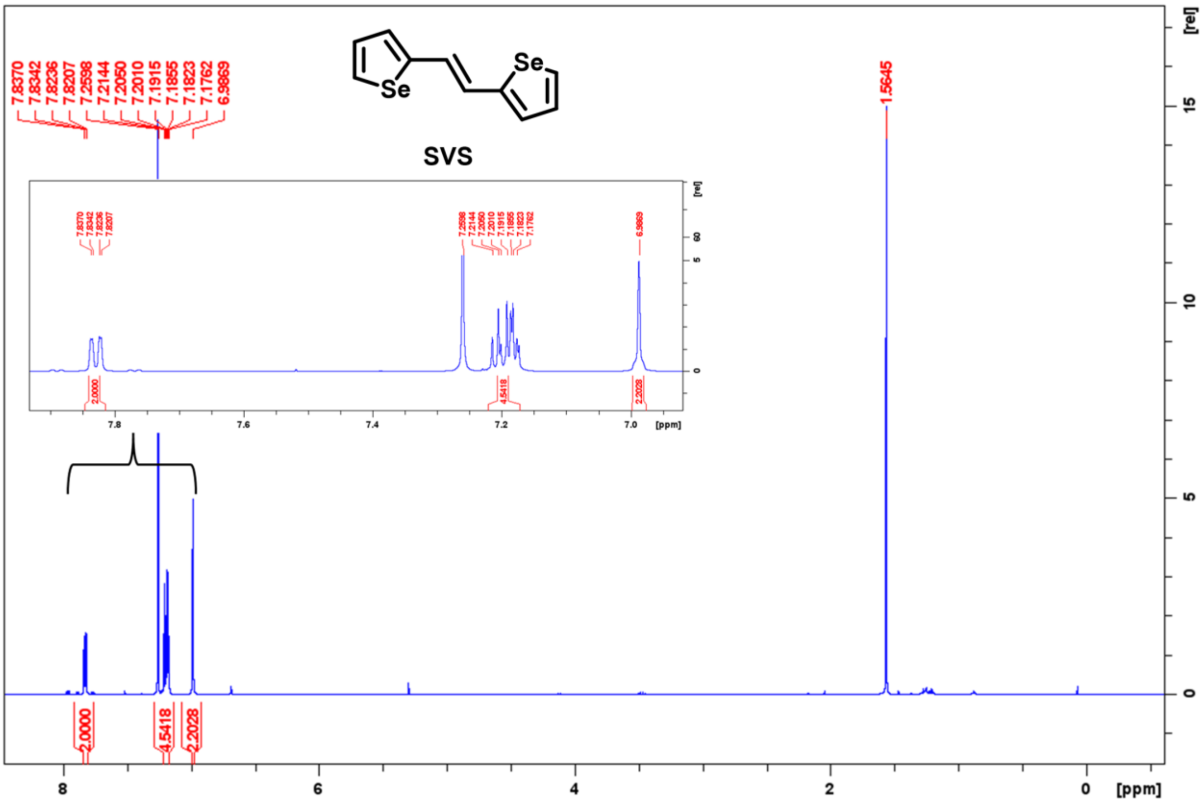

1.2.2. SVS-Sn

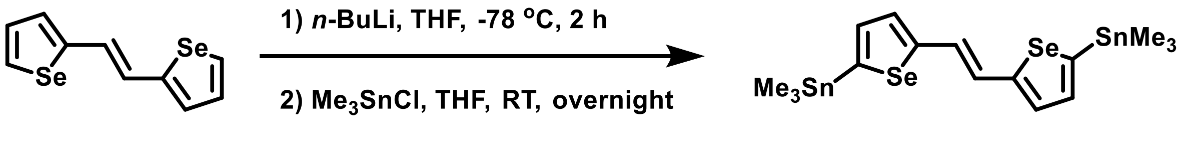

SVS

SVS-Sn

SVS (0.35 g, 1.23 mmol) was dissolved in anhydrous THF (10 mL) under N_2 atmosphere, and then cooled to -78 °C. A 2.5 M solution of *n*-BuLi in *n*-hexane (1.18 mL, 2.94 mmol) was added

drop-wise to the reaction mixture, and stirred for 2 h at this temperature, and then for 1 h at room temperature to give a green suspension. A 1 M solution of trimethyltin chloride in THF (2.94 mL, 2.94 mmol) was added slowly to the reaction mixture at -78 °C, and then the solution was stirred at room temperature overnight. After removal of the solvent, the crude product was purified by column chromatography using neutral aluminum oxide (including 10% K_2CO_3) and *n*-hexane/CH_2Cl_2 (5:1) as eluent. Then the product was further purified by recrystallization from MeOH at 50 °C to yield yellow crystals of **SVS-Sn** (0.45 g, 60%). ^{1}H NMR (400 MHz, $CDCl_3$): δ 7.35 (d, J = 3.54 Hz, 2H), 7.25 (m, 2H), 7.02 (s, 2H), 0.37 (s, 18H) ppm.

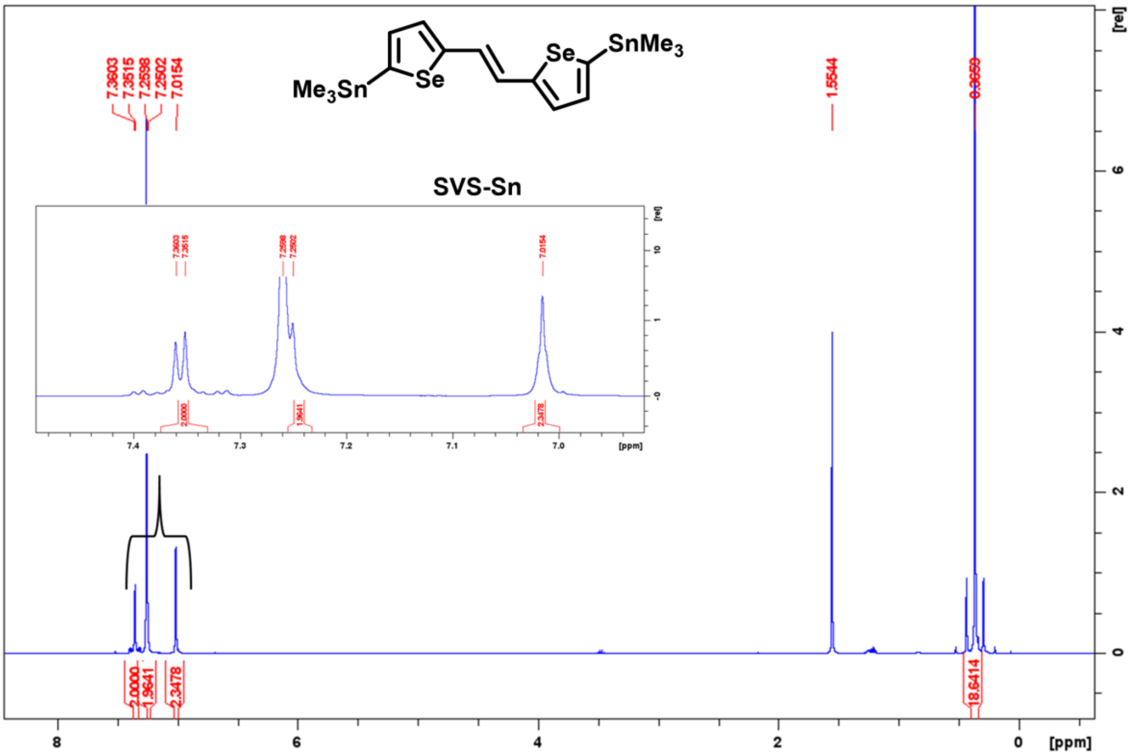

1.2.3. Se-COOH

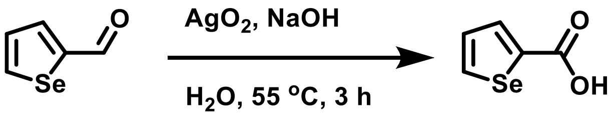

Se-CHO **Se-COOH**

A solution of $AgNO_3$ (3.20 g, 29.5 mmol) in H_2O (30 mL) was added to a solution of NaOH (1.39 g, 31.6 mmol) in H_2O (15 mL) to prepare Ag_2O. The precipitated AgO_2 was isolated by

filtration and then washed with H_2O. The fresh AgO_2 was added to a solution of NaOH (2.29 g, 57.6 mmol) in 30 mL H_2O, and the mixture was stirred for 15 min at room temperature. **Se-CHO** (3.00 g, 18.9 mmol) was added to the mixture of AgO_2 and NaOH, and the reaction mixture was stirred at room temperature for 30 min followed by heating to 50 °C for 3 h. A crystalline precipitate was filtered off and washed with water. The filtrate and washing solutions were combined and poured into ice-cold 10% HCl solution. The solution was extracted with ethyl acetate, and then the organic extract was washed with water and brine, and then dried over $MgSO_4$. The solvent was removed *in vacuo* and the residue was further dried to give **Se-COOH** (3.00 g, 91%) as a pale-yellow solid. ^1H NMR (400 MHz, $CDCl_3$): δ 8.39 (dd, J = 6.42 Hz, J = 4.51 Hz, 1H), 8.15–8.14 (dd, J = 4.82 Hz, J = 2.80 Hz, 1H), 7.40 (t, J = 3.13 Hz, 1H) ppm.

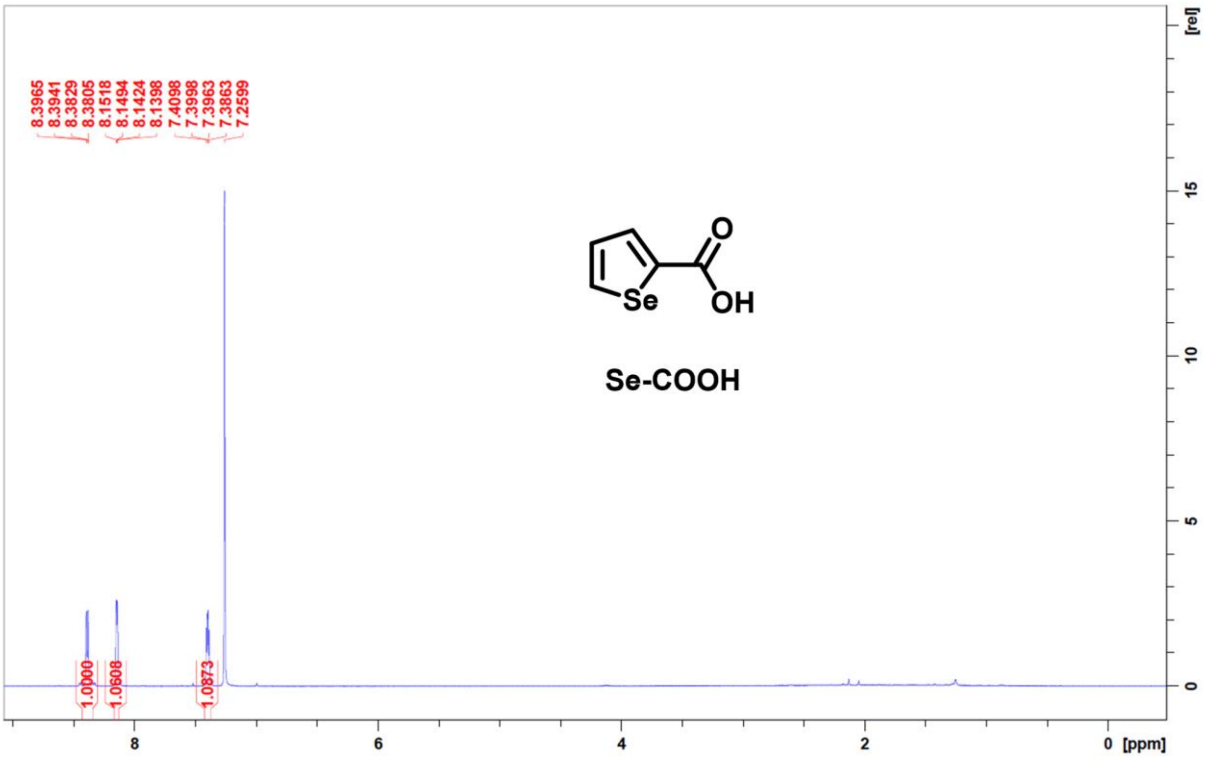

1.2.4. FSe-COOH

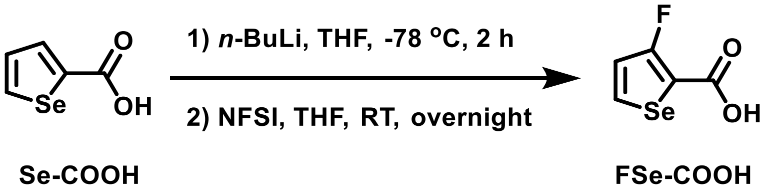

Se-COOH (3.00 g, 17.1 mmol) was dissolved in anhydrous THF (75 mL) under N_2 atmosphere, and then cooled to -78 °C. *n*-BuLi in *n*-hexane (17.0 mL, 42.5 mmol) was added slowly into the reaction solution at this temperature, and the mixture was stirred for 2 h at the same temperature. Degased *N*-fluorobenzenesulfonimide solution (8.10 g, 25.7 mmol) in anhydrous THF was slowly added into the reaction mixture at -78 °C and the mixture was stirred for 4 h at the same temperature. The reaction mixture was warmed to room temperature and stirred overnight. After acidification using 6 M HCl (10 mL, pH<7), the reaction mixture was extracted with ethyl acetate. The organic phase was washed with brine and dried over $MgSO_4$. The solvent was removed under reduced pressure and the crude product was purified by silica gel column chromatography with *n*-hexane/ethyl acetate (20% ethyl acetate) with 1.5% acetic acid. Finally, **FSe-COOH** was obtained as a yellow solid (1.60 g, 48%). ^{1}H NMR (400 MHz, $CDCl_3$): δ 8.20 (dd, J = 6.1 Hz, J = 4.0 Hz, 1H), 7.25 (d, 1H) ppm. ^{19}F NMR (376 MHz, $CDCl_3$): δ – 104.3 ppm.

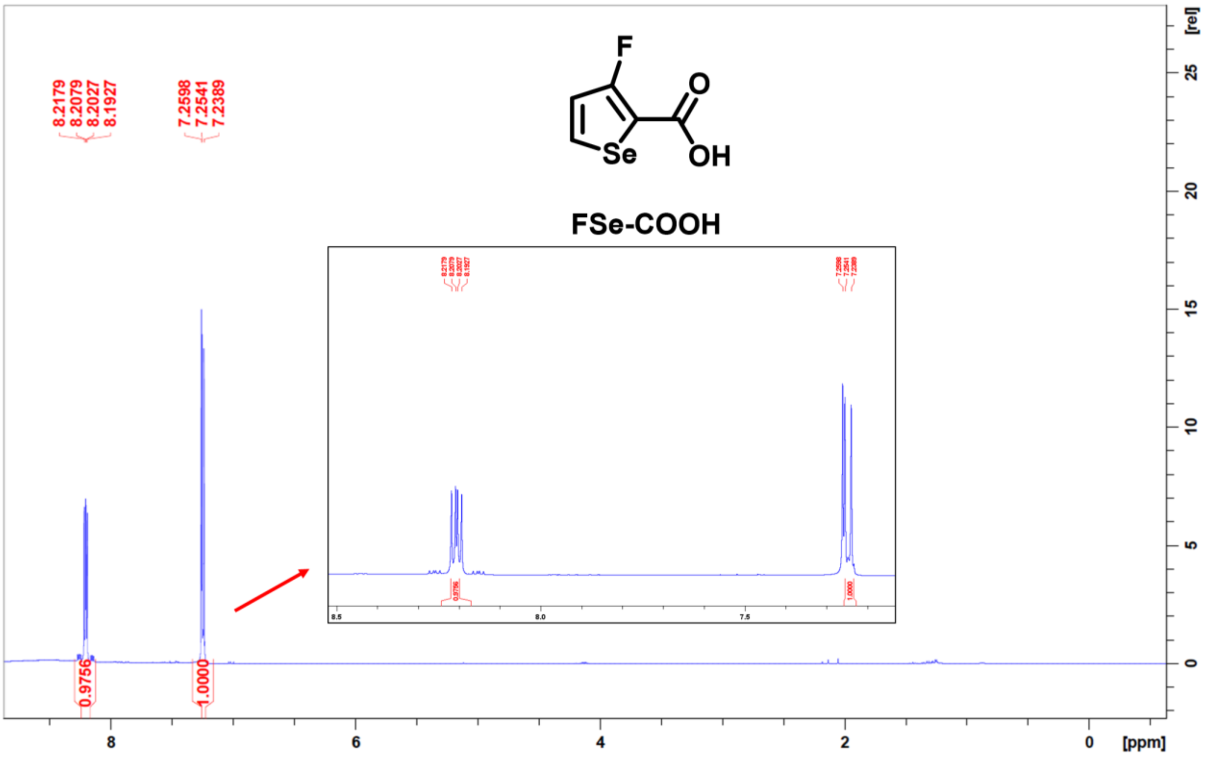

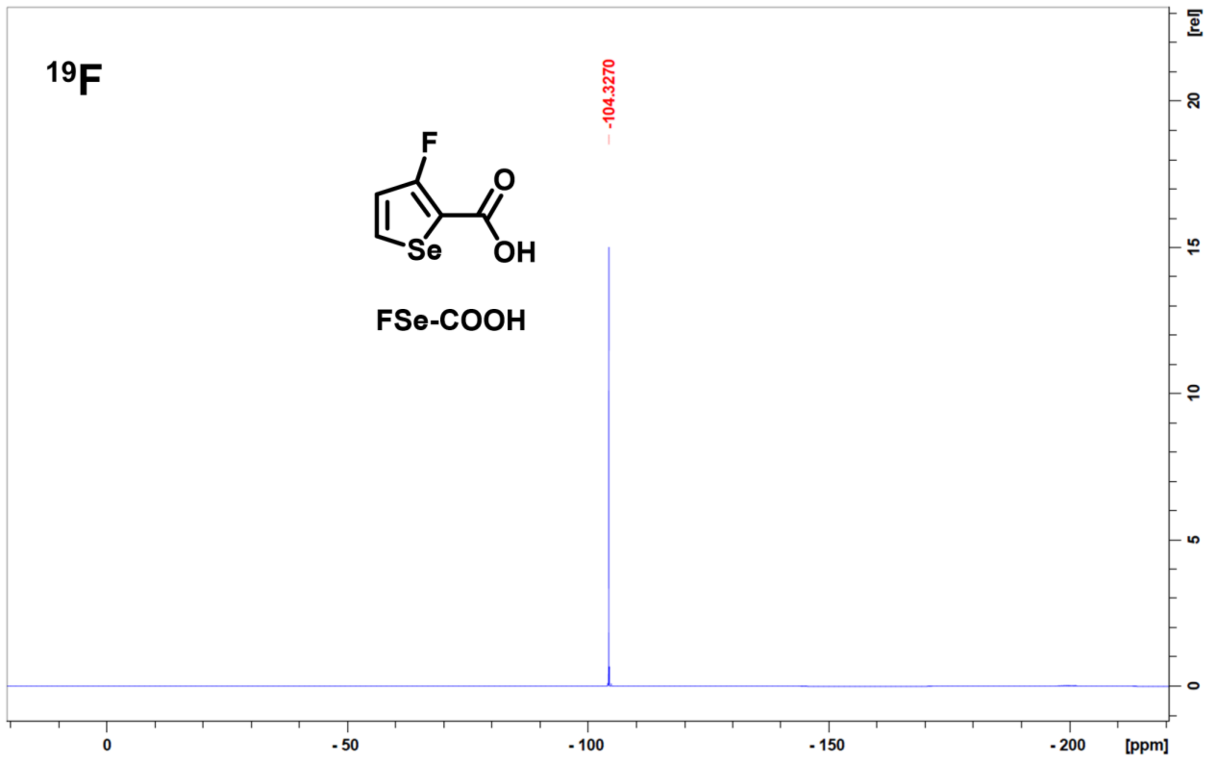

1.2.5. FSe-COOMe

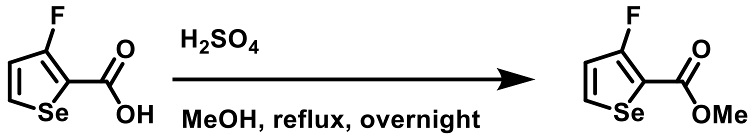

FSe-COOH (1.57 g, 8.13 mmol) was dissolved in MeOH (80 mL) and concentrated H_2SO_4 (1.5 mL) was added into the solution. The mixture was heated under reflux overnight. The reaction mixture was cooled to room temperature, and 15% NaOH solution in H_2O was added into the mixture for neutralization (pH>7). The reaction mixture was extracted with ethyl acetateand the organic phase was washed with brine and H_2O, then dried over $MgSO_4$. Finally, **FSe-COOMe** (R_f: 0.38 on TLC under *n*-hexane/ethyl acetate (10% ethyl acetate) was obtained as pale-yellow solid (1.50 g, 89%). ^{1}H NMR (400 MHz, $CDCl_3$): δ 8.09 (d, J = 6.11 Hz, 1H), 7.22–7.20 (d, J = 6.1 Hz, 1H), 3.87 (s, 3H) ppm. ^{19}F NMR (376 MHz, $CDCl_3$): δ – 107.2 ppm.

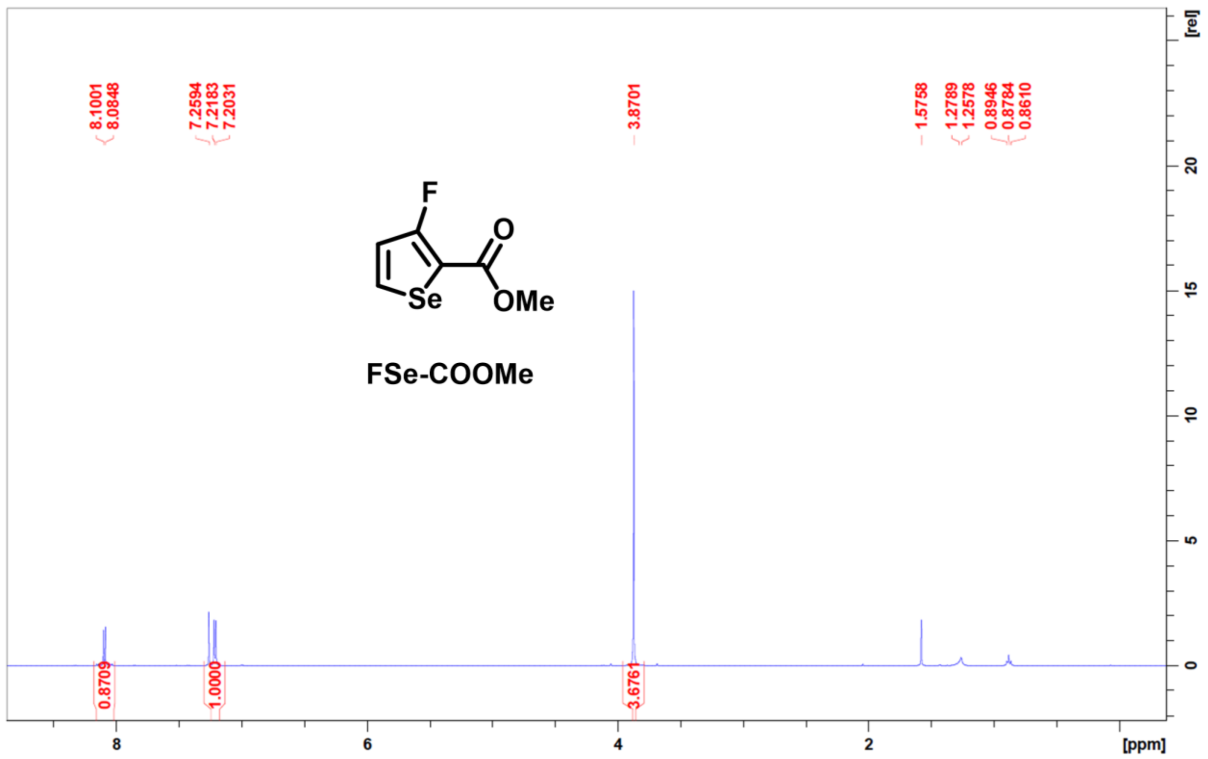

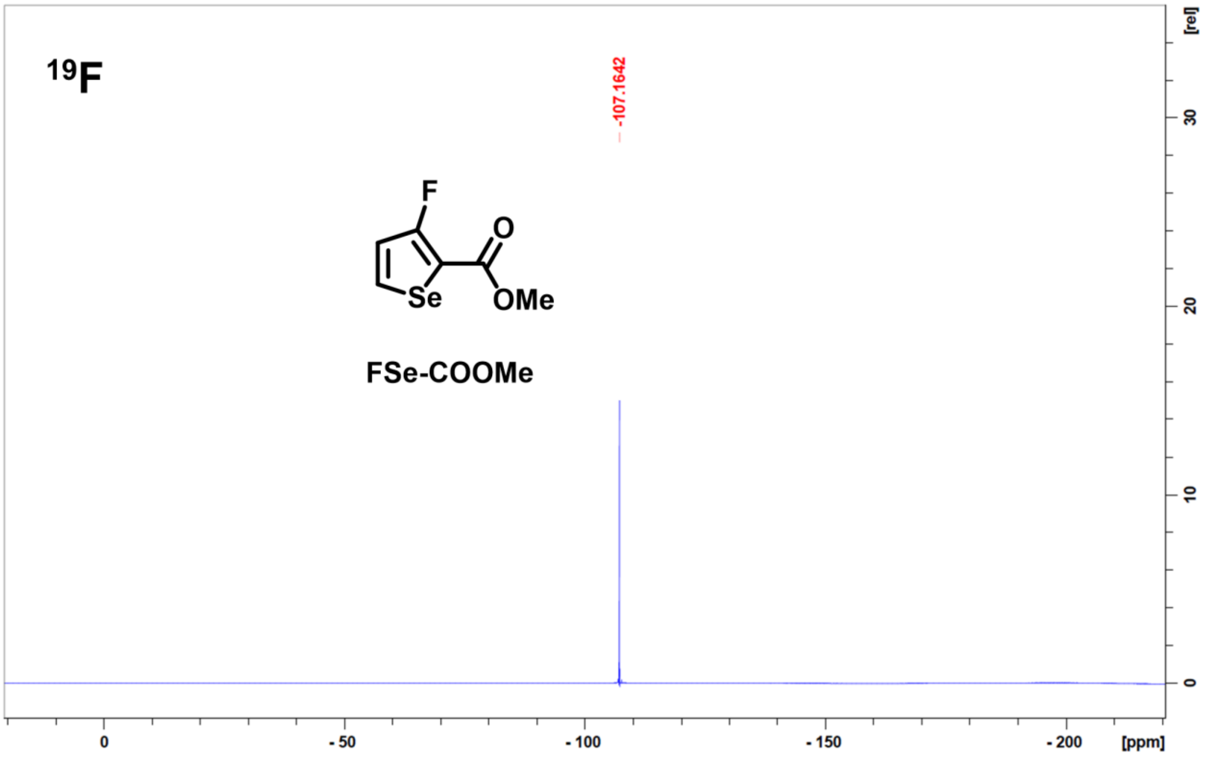

1.2.6. FSe-CH2OH

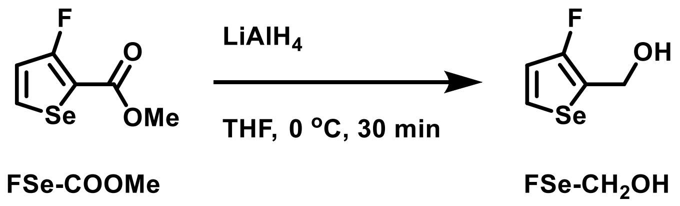

A 2.4 M solution of $LiAlH_4$ (3.32 mL, 7.96 mmol) in THF was added drop-wise to a solution of **FSe-COOMe** (1.50 g, 7.24 mmol) in anhydrous THF (30 mL) at -10 °C, and the mixture was stirred for 30 min at this temperature. After slow addition of H_2O (0.5 mL) at -10 °C, 15% NaOH aqueous solution (0.5 mL) and H_2O (1.5 mL) were sequentially added into the mixture. The mixture was warmed to room temperature, and stirred for 30 min. $MgSO_4$ was added into the mixture to dry the mixture. After filtration, the filtrate was concentrated and the crude product was purified by silica gel column chromatography with *n*-hexane/ethyl acetate (5-15% ethyl acetate). Finally, **FSe-CH$_2$OH** (R_f: 0.48 on TLC under *n*-hexane/ethyl acetate (30% ethyl acetate)) was obtained as a colorless oil (1.10 g, 85%). ^{1}H NMR (400 MHz, $CDCl_3$): δ 7.80 (d, J = 6.03 Hz, 1H), 7.10 (d, J = 6.02 Hz, 1H), 4.83 (s, 2H), 1.93 (s, 1H) ppm. ^{19}F NMR (376 MHz, $CDCl_3$): δ -126.3 ppm.

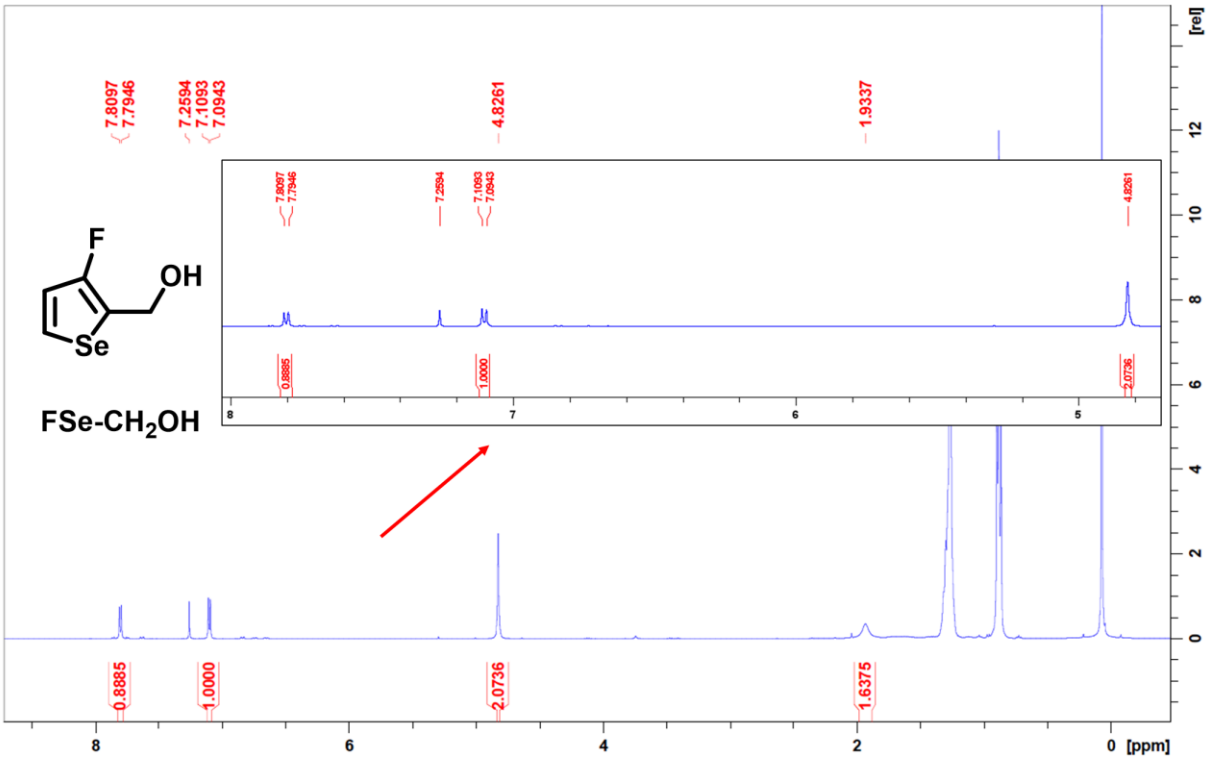

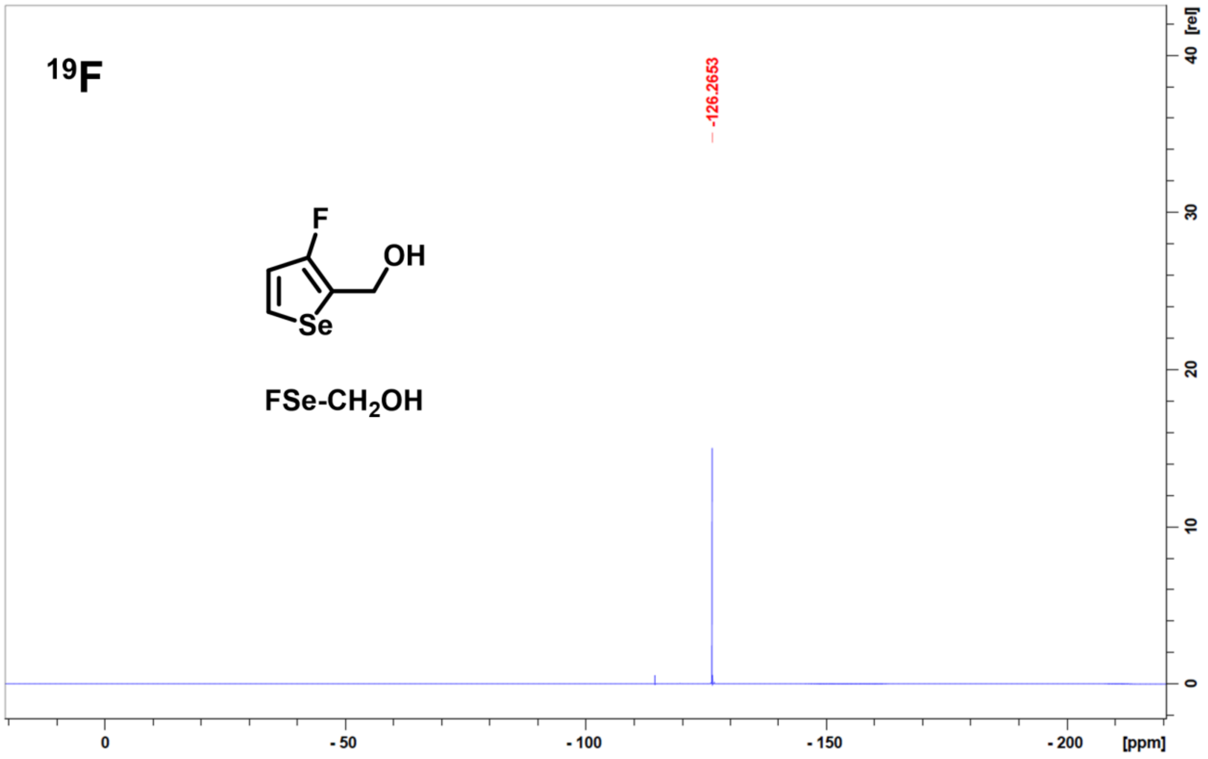

1.2.7. FSe-CHO

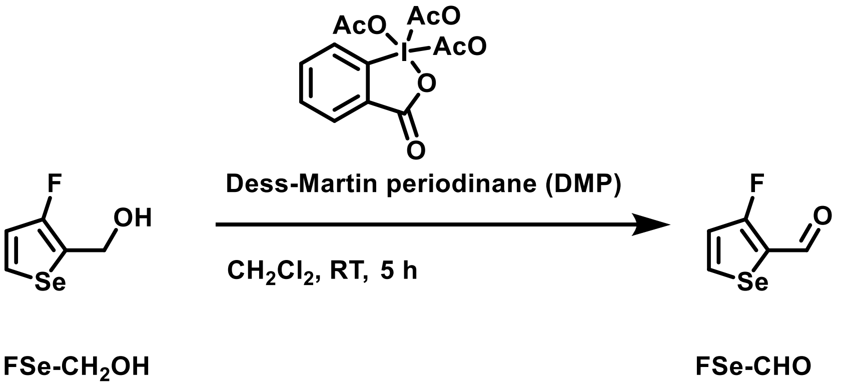

FSe-CH2OH (1.00 g, 5.59 mmol) and DMP (3.08 g, 7.27 mmol) were dissolved in CH_2Cl_2 (40 mL), and the mixture was stirred for 5 h at room temperature. The solvent was carefully evaporated using a rotary evaporator under reduced pressure (the product is volatile). The crude product was purified using column chromatography with *n*-hexane/ethyl acetate (ethyl acetate 1-10%). The solvent was carefully removed *in vacuo*(product is volatile) to give **FSe-CHO** (R_f: 0.43 on TLC under *n*-hexane/ethyl acetate (10% ethyl acetate)) as a colorless oil

(0.90 g, 90%). ^{1}H NMR (400 MHz, $CDCl_3$): δ 9.96 (s, 1H), 8.34 (d, J = 6.00 Hz, 1H), 7.24 (d, 1H) ppm. ^{19}F NMR (376 MHz, $CDCl_3$): δ-113.4 ppm.

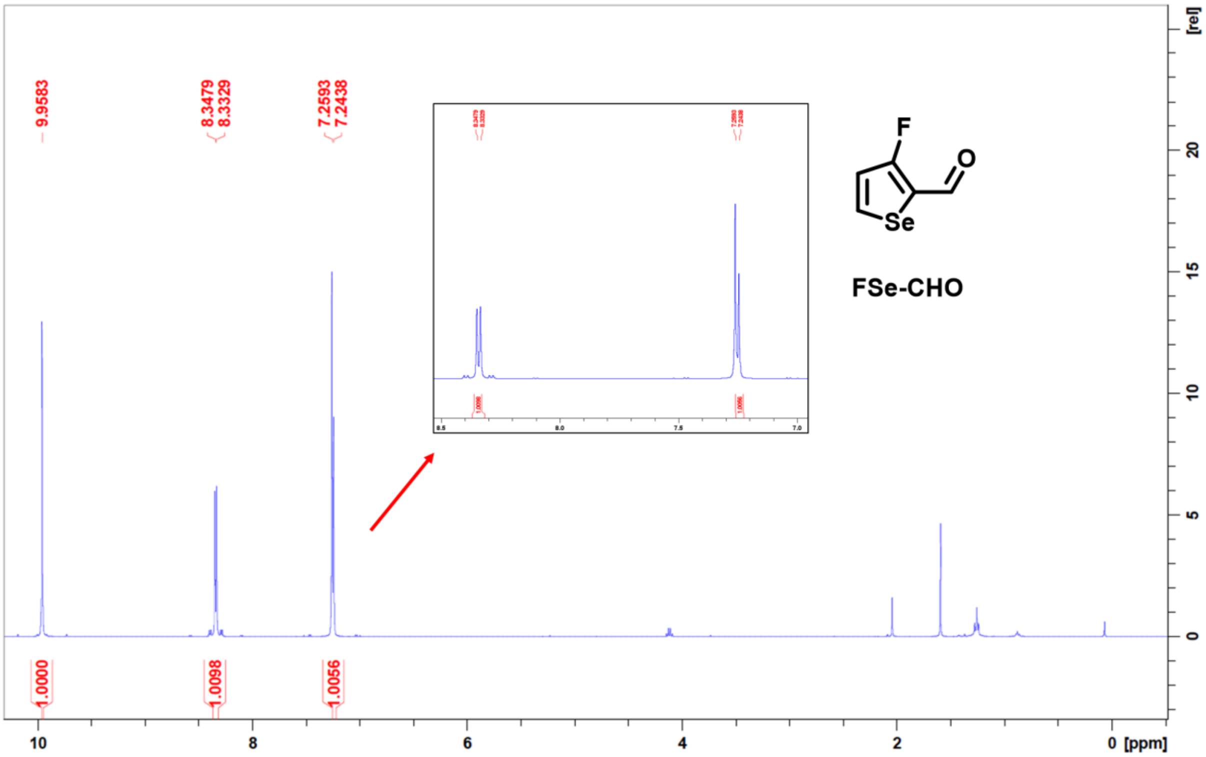

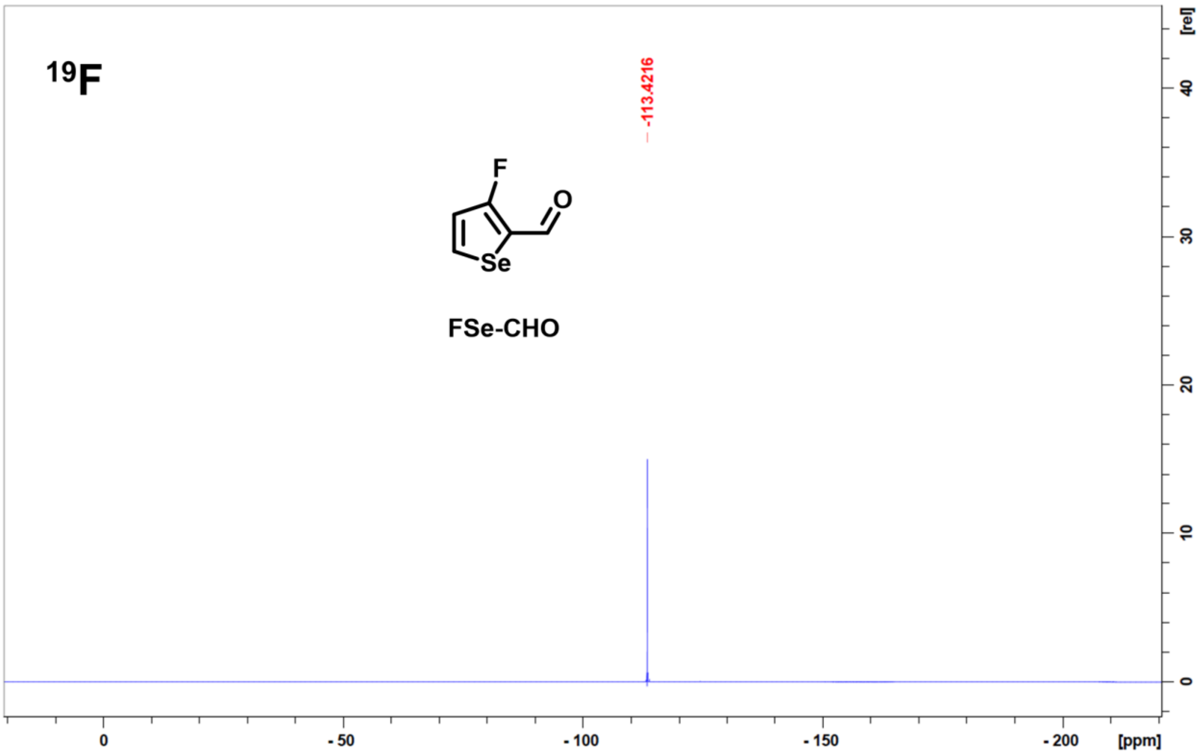

1.2.8. FSVS

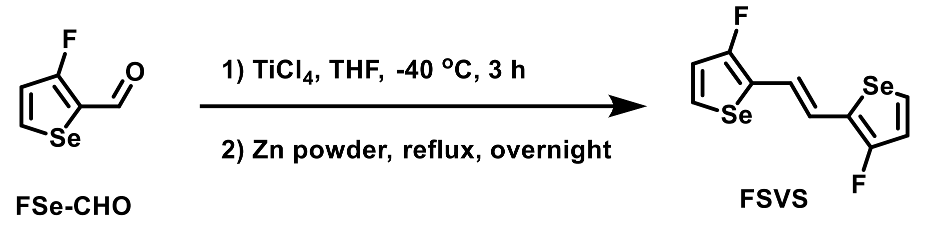

A 1 M solution of $TiCl_4$ in CH_2Cl_2 (7.63 mL, 7.63 mmol) was added drop-wise to a solution of **FSe-CHO** (0.90 g, 5.09 mmol) in THF (30 mL) at -40 °C under N_2 atmosphere, and the mixture was stirred at this temperature for 3 h. Zn powder (1.02 g, 15.3 mmol) was added portion-wised over a period for 30 min at -40 °C. The mixture was stirred at this temperature for 30 min, and then was heated under reflux overnight. The reaction mixture was quenched by addition of ice-cood H_2O (50 mL). The precipitate was isolated by filtration and dried. The residual solid was dissolved in CH_2Cl_2 (50 mL) and the remaining solids were removed by filtration. The solvent was removed under reduced pressure and the crude product was purified by column chromatography over neutral aluminum oxide with *n*-hexane/CH_2Cl_2 (from 20:1 to 5:1) as eluent. **FSVS** (R_f: 0.58 on TLC under *n*-hexane/CH_2Cl_2 (20% CH_2Cl_2)) was obtained as pale-yellow solid (0.37 g, 45%). ^{1}H NMR (400 MHz, $CDCl_3$): δ 7.66 (d, J = 6.1 Hz, 2H), 7.10 (d, J = 6.1 Hz), 6.89 (s, 2H) ppm. ^{19}F NMR (376 MHz, $CDCl_3$): δ-123.7 ppm.

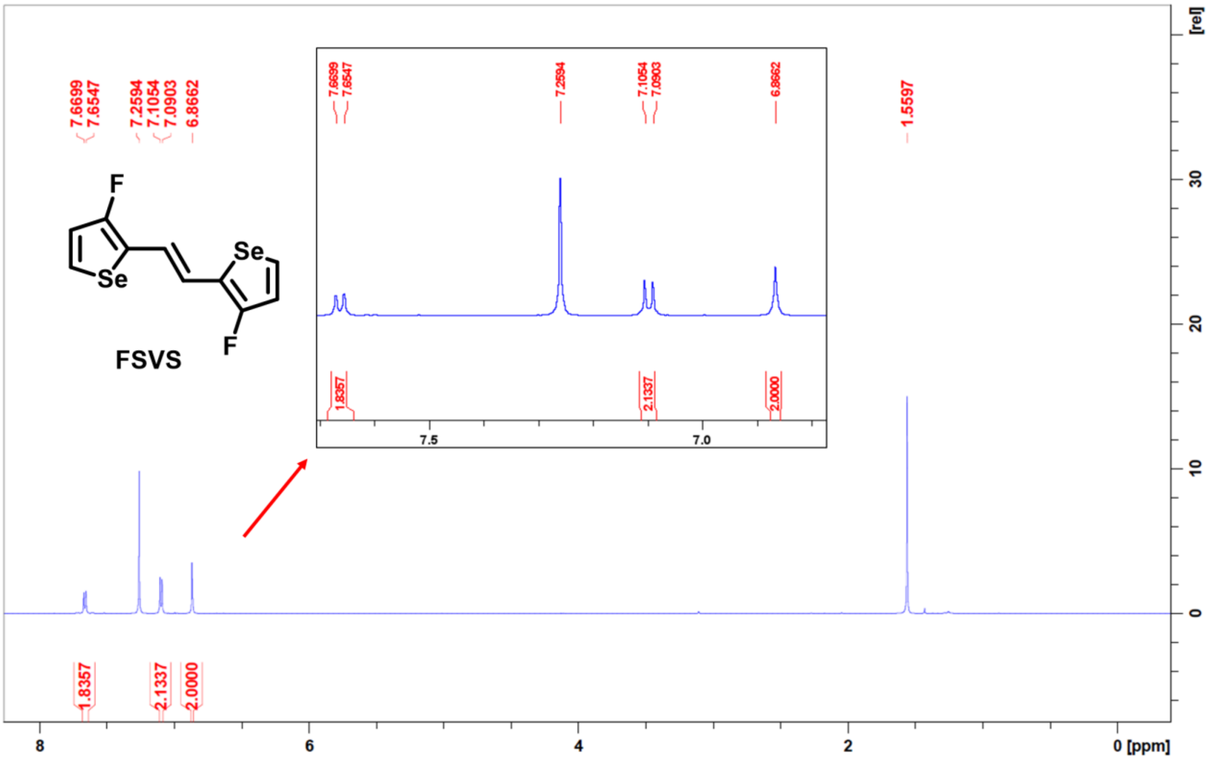
7.6699
7.6547
7.2594
7.1054
7.0903
6.8662
1.5597
FSVS
1.8357
2.1337
2.0000
7.5
7.0
8
6
4
2
0 [ppm]
[rel]

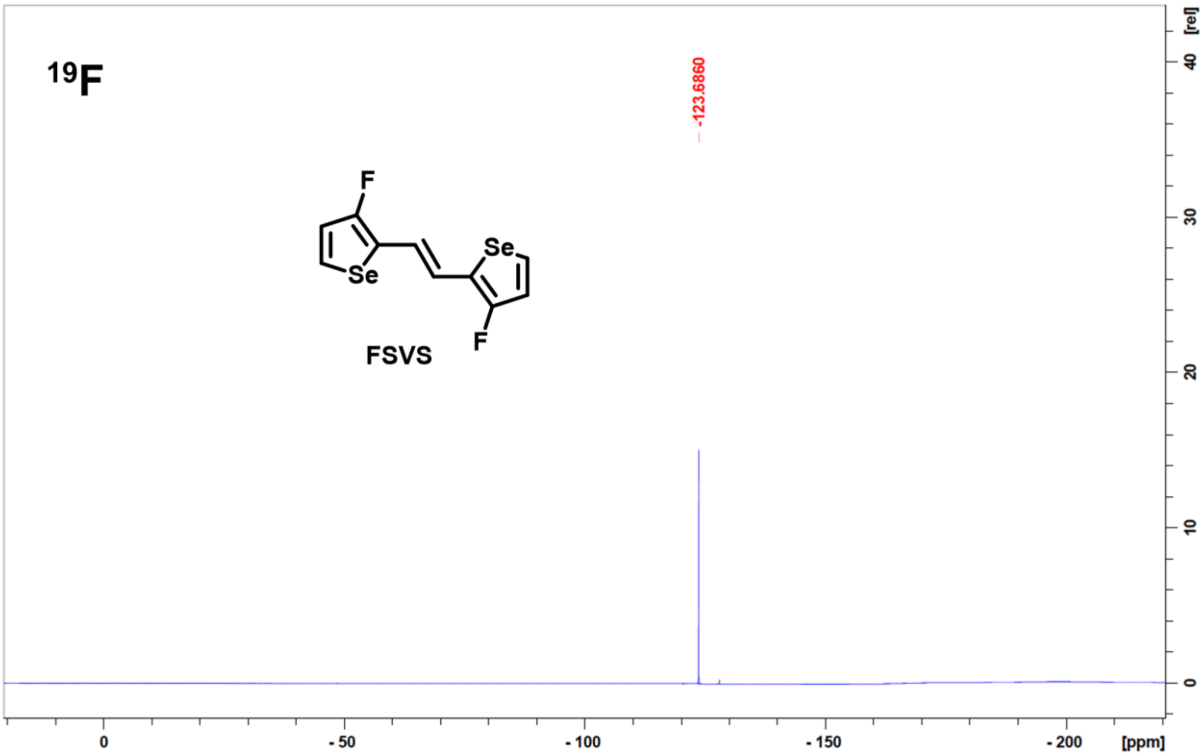
19F
-123.6860
FSVS
0
- 50
- 100
- 150
- 200
[ppm]
[rel]

1.2.9. FSVS-Sn

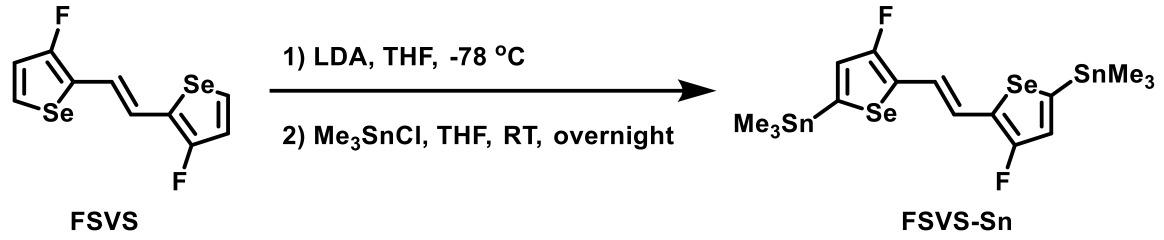

FSVS (0.30 g, 0.93 mmol) was dissolved in anhydrous THF (10 mL) under N_2 atmosphere, and then cooled to -78 °C. A 2.0 M solution of LDA in THF (1.16 mL, 2.33 mmol) was added drop-wise to the reaction mixture, which was stirred at this temperature for 3 h. A 1 M solution of trimethyltin chloride in THF (2.33 mL, 2.33 mmol) was added slowly to the reaction mixture at -78 °C, and then the mixture was stirred for 3h at this temperature. The mixture was warmed to room temperature, and then stirred overnight. After removal of the solvent, the crude product was purified by column chromatography over neutral aluminum oxide powder (including 10% K_2CO_3) using *n*-hexane as eluent. The product was further purified by recrystallization from MeOH at 50 °C to yield orange crystals of **FSVS-Sn** (0.11 g, 19%). ^{1}H NMR (400 MHz, $CDCl_3$): δ 7.24 (s, 2H), 6.83 (s, 2H), 0.37 (s, 18H) ppm. ^{19}F NMR (376 MHz, $CDCl_3$): δ-125.5 ppm.

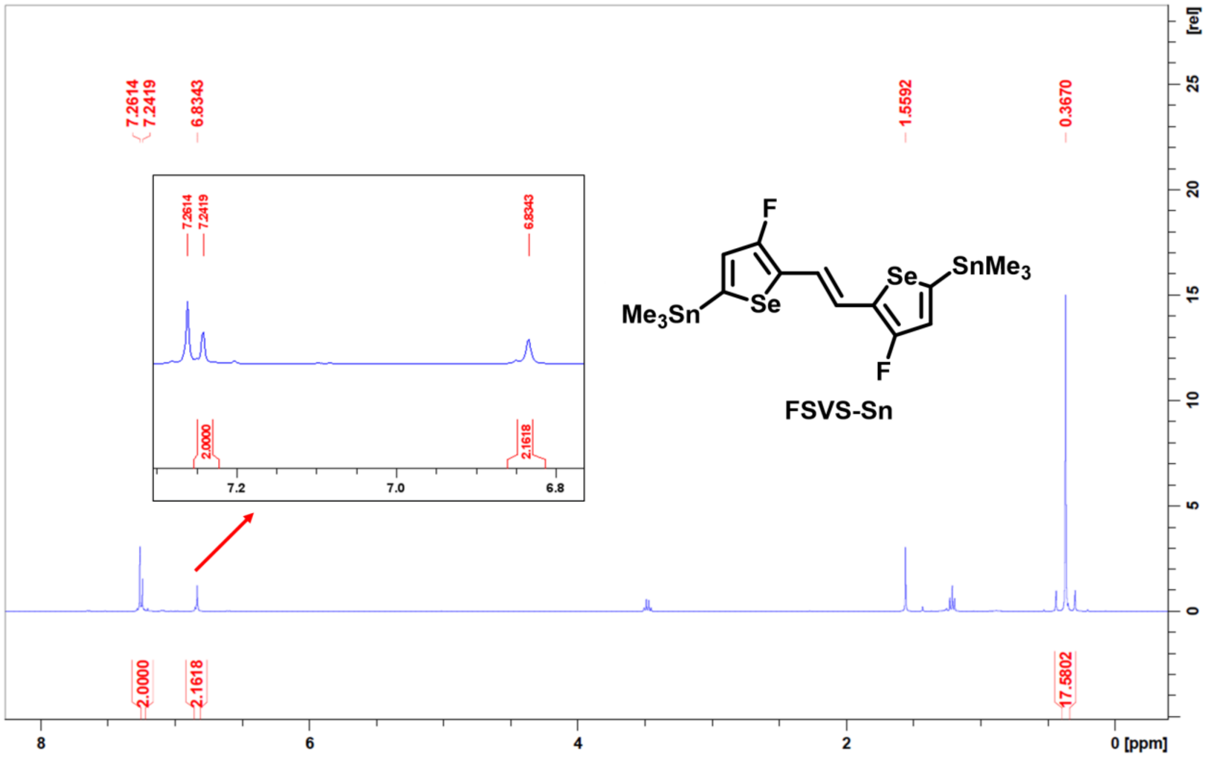

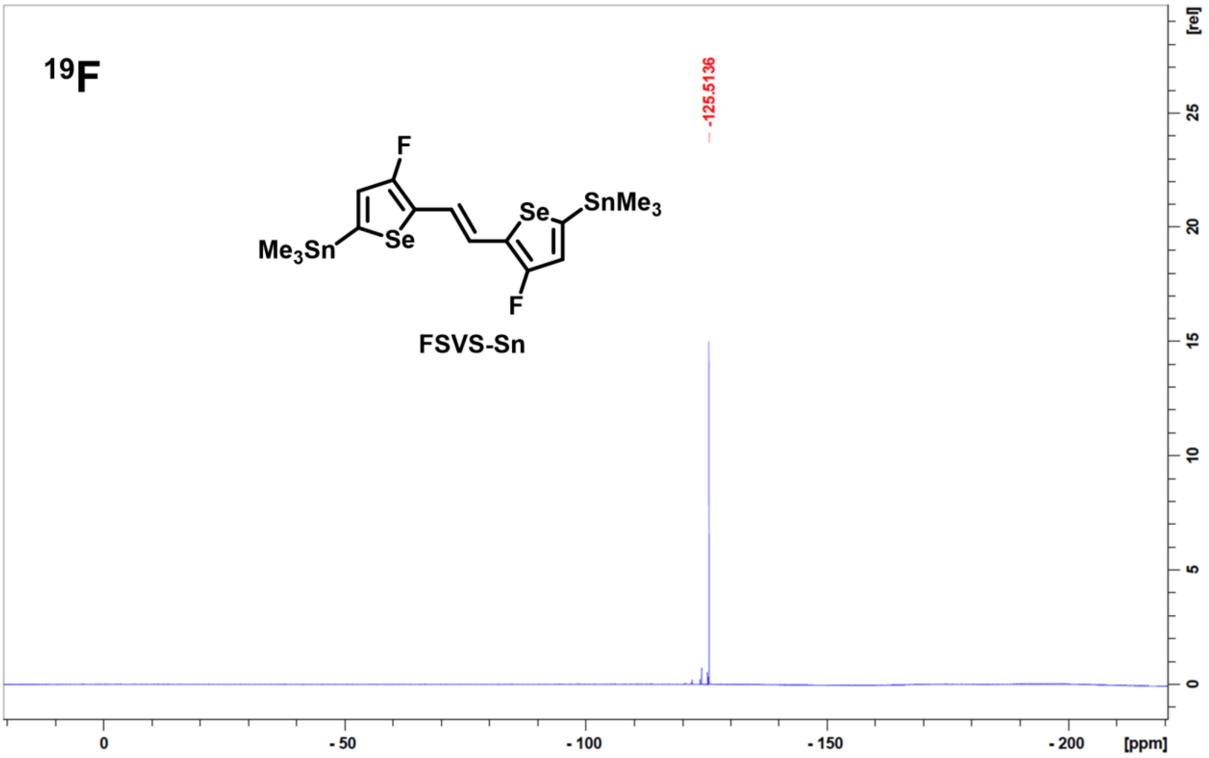

1.2.10. NDIEG7-Br

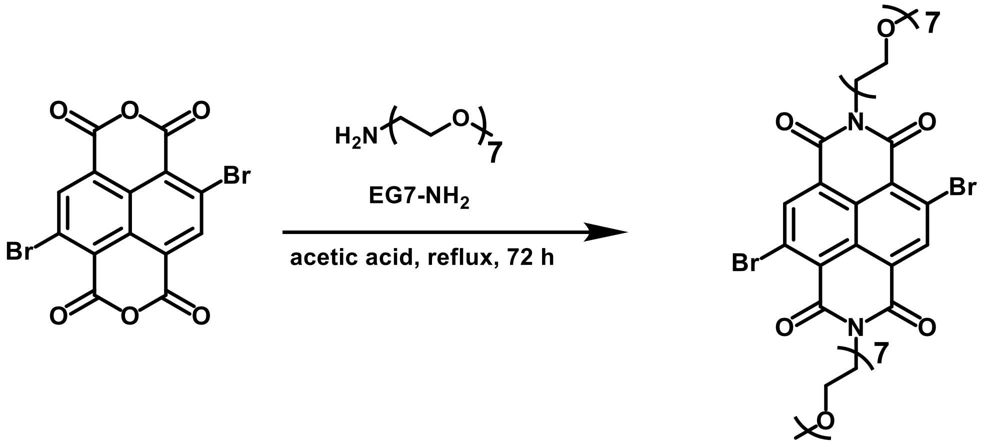

NDA-Br (554 mg, 1.30 mmol) and EG7-NH_2 (1.00 g, 2.95 mmol) were dissolved in AcOH (70 mL) in a 2-neck round-bottom flask under N_2 atmosphere. Then, the mixture was heated under reflux at 120 °C for 72 h until NDA-Br was completely dissolved in solvent. The mixture was concentrated using a rotary evaporator under reduced pressure at 70 °C. The crude product was

purified by column chromatography with ethyl acetate/MeOH (10% MeOH) to yield **NDIEG7-Br** as a yellow/red sticky solid (1.00 g, 72%). ^{1}H NMR (400 MHz, $CDCl_3$): δ 8.98 (s, 2H), 4.46 (t, J = 5.82 Hz, 4H), 3.84 (t, J = 5.82 Hz, 4H), 3.71–3.69 (m, 4H), 3.65–3.60 (m, 40H), 3.55–3.53 (m, 4H), 3.37 (s, 6H) ppm.

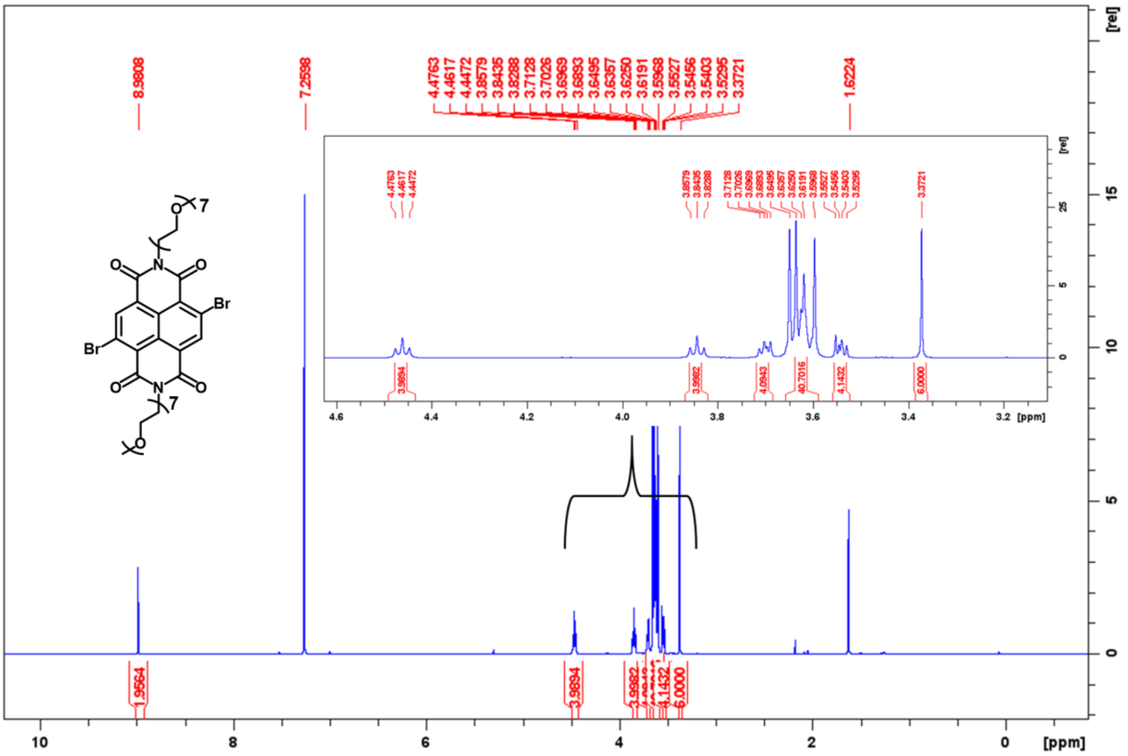

1.3. Polymerization

1.3.1. General process for polymerization

Brominated acceptors (**NDIOD** or **NDIEG7**, 1.0 eq.) and stannylated donors (**SVS-Sn** or **FSVS-Sn**, 1.0 eq.) were placed in a 3-necked round-bottom flask. The reaction flask was subjected to 3 times of vacuum-pumping/purging cycles with N_2 gas. Then, anhydrous chlorobenzene was added using a syringe. After addition of $Pd(0)(PPh_3)_4$ catalyst (0.5 mol%), the reaction mixture was stirred at 130 °C for 18 h. After the polymerization, 2-bromothiohene (1 eq.) and 2-(tributylstannyl)thiophene (1.0 eq.) were injected as the end-capping reagents were injected to the reaction mixture and the reaction was continued at the same temperature for 3 h. The mixture was cooled to room temperature and added slowly into a large volume of

methanol to precipitate the solid polymer. The precipitated polymer was isolated by filtration and purified by Soxhlet using MeOH, acetone and chloroform, sequentially. Finally, the chloroform fraction including purified polymer was concentrated under reduced pressure and the residue was precipitated into MeOH to yield the polymer powders after filtration and vacuum-drying.

For P(NDIOD-FSVS) and P(NDIEG7-FSVS):

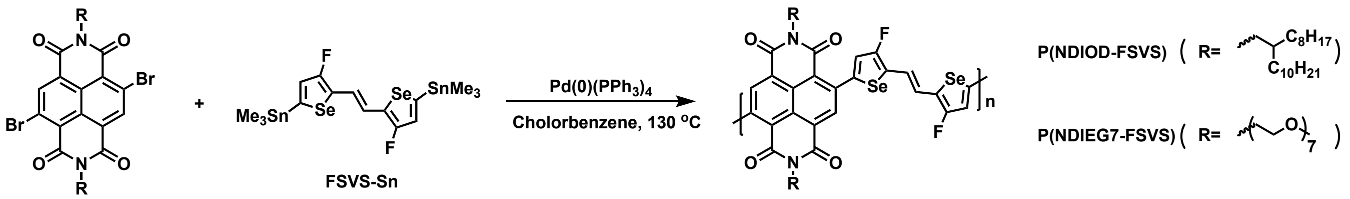

1.3.2. P(NDIOD-FSVS)

NDIOD (67 mg, 77 µmmol), **FSVS-Sn** (50 mg, 77 µmol), Pd(0)(P(Ph$_3$)$_4$ (4.5 mg, 4 µmol), anhydrous chlorobenzene (6.0 mL), 2-bromothiophene (0.01 mL), tributyl(thiophene-2-yl)stannane (0.03 mL)

^{1}H NMR (400 MHz, CDCl$_3$): δ 8.78 (br, 2H, NDI unit), 7.38 (br, 2H, FSVS unit), 7.05 (br, 2H, FSVS unit), 4.11 (br, 4H, alkyl), 1.96–1.67 (br, 2H, alkyl), 1.43–1.25 (br, 80H, alkyl), 1.00–0.86 (m, 12H, alkyl) ppm. *Mn*= 10956 g/mol, *Mw*= 25053 g/mol, PDI =2.29.

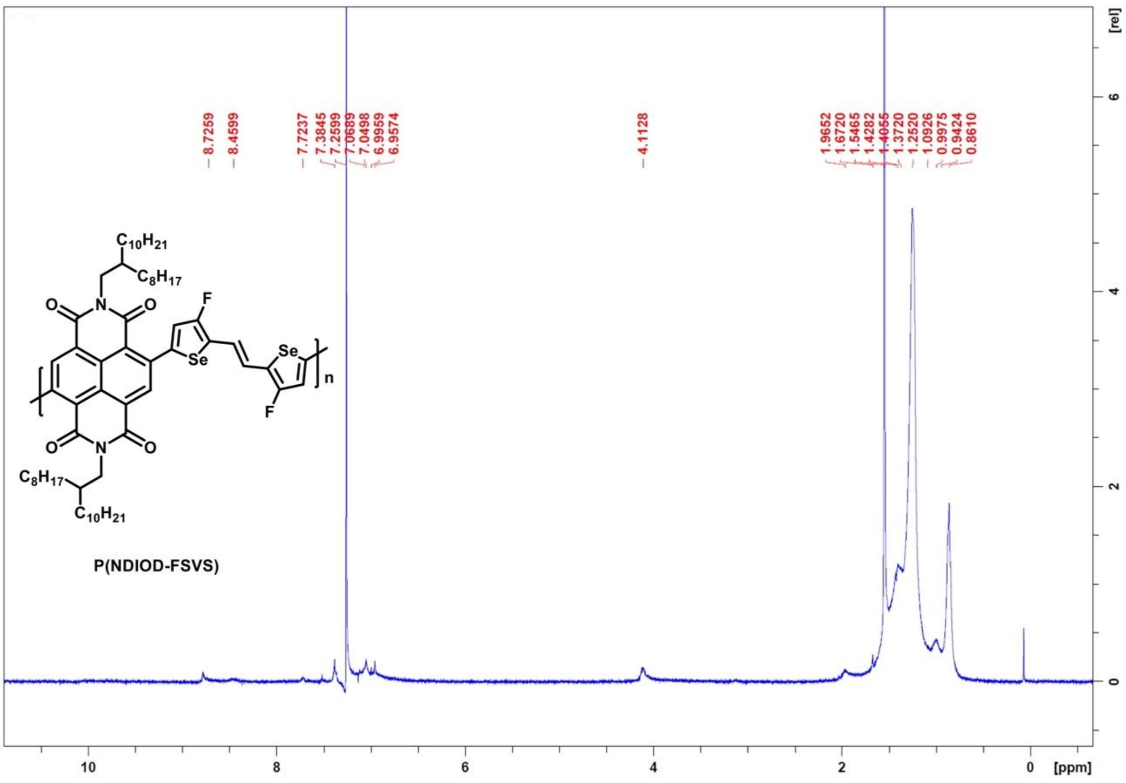

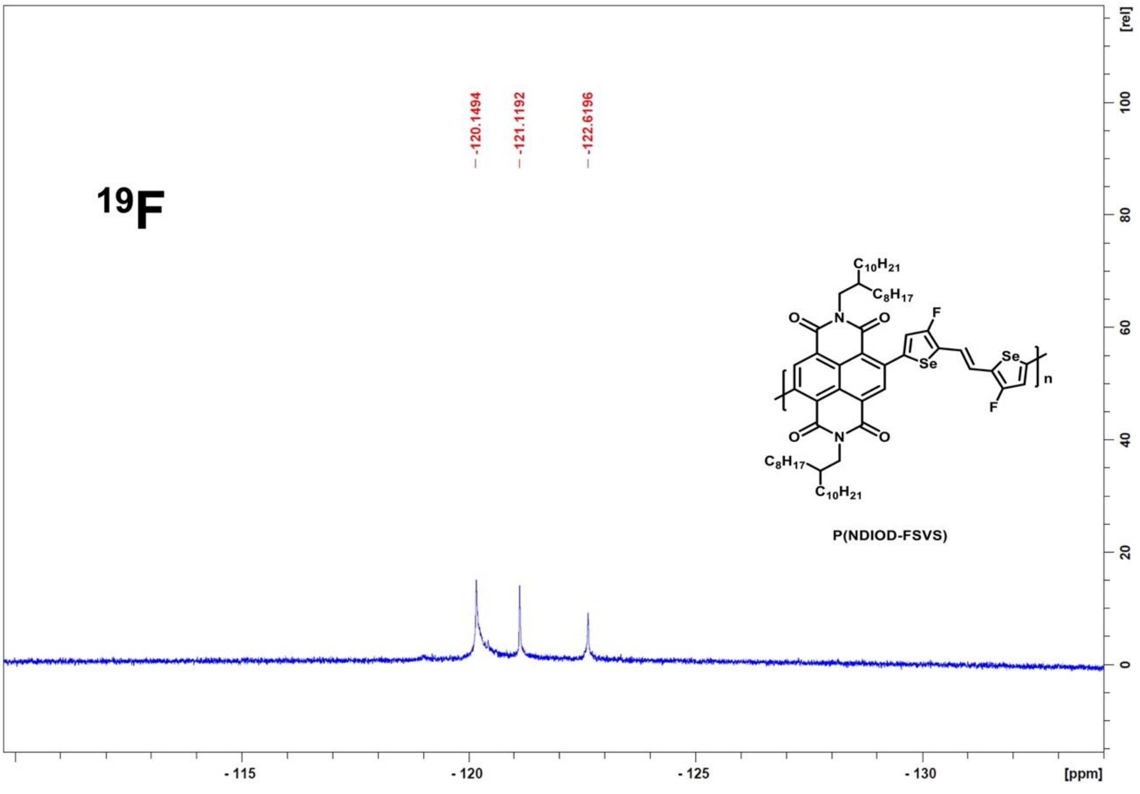

1.3.3. P(NDIEG7-FSVS)

NDIEG7 (83 mg, 77 µmmol), **FSVS-Sn** (50 mg, 77 µmol), Pd(0)(P(Ph_3)$_4$ (4.5 g, 4.5 µmol), anhydrous chlorobenzene (6.0 mL), 2-bromothiophene (0.01 mL), tributyl(thiophene-2-yl)stannane (0.03 mL).

^{1}H NMR (400 MHz, $CDCl_3$): δ 8.72–8.32 (br, 2H, NDI unit), 7.67–7.30 (br, 2H, FSVS unit), 7.05–6.89 (br, 2H, FSVS unit), 4.37 (s, 4H, alkyl), 3.76–3.38(m, 44H, OCH_2CH_2 for EG chain), 3.30 (s, 6H OCH_3 for EG chain) ppm. *Mn*= 6569 g/mol, *Mw*= 16558 g/mol, PDI =2.52.

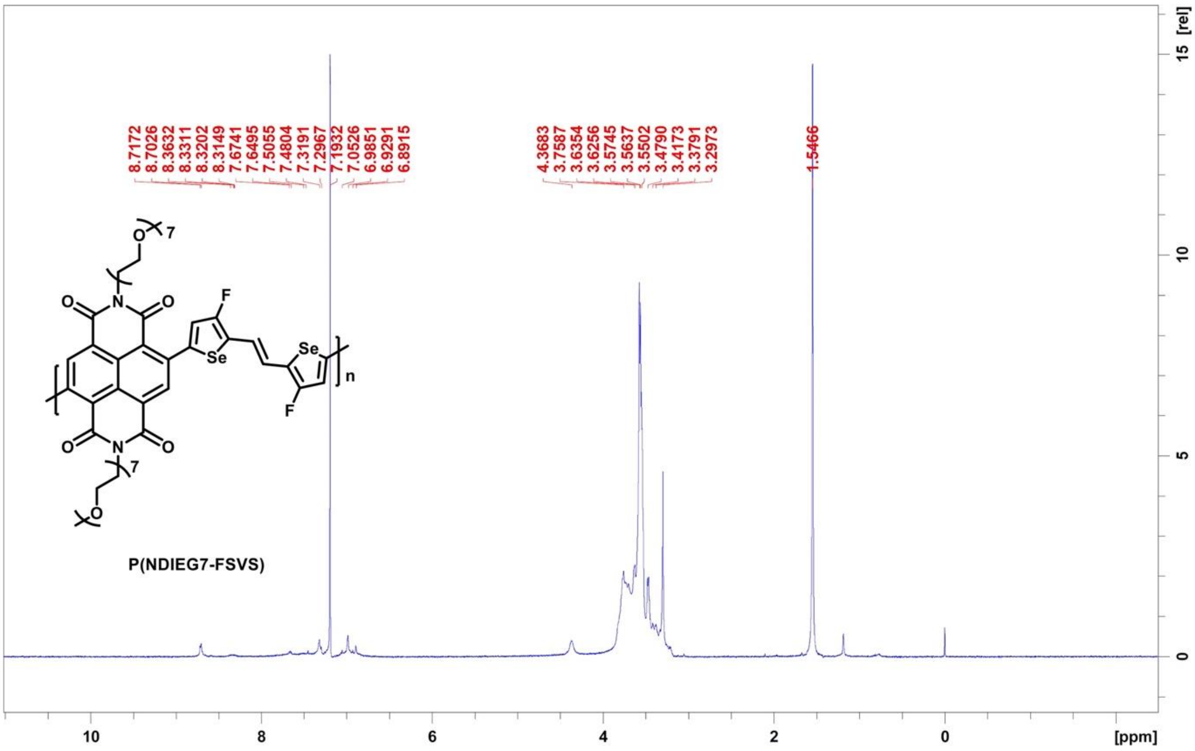

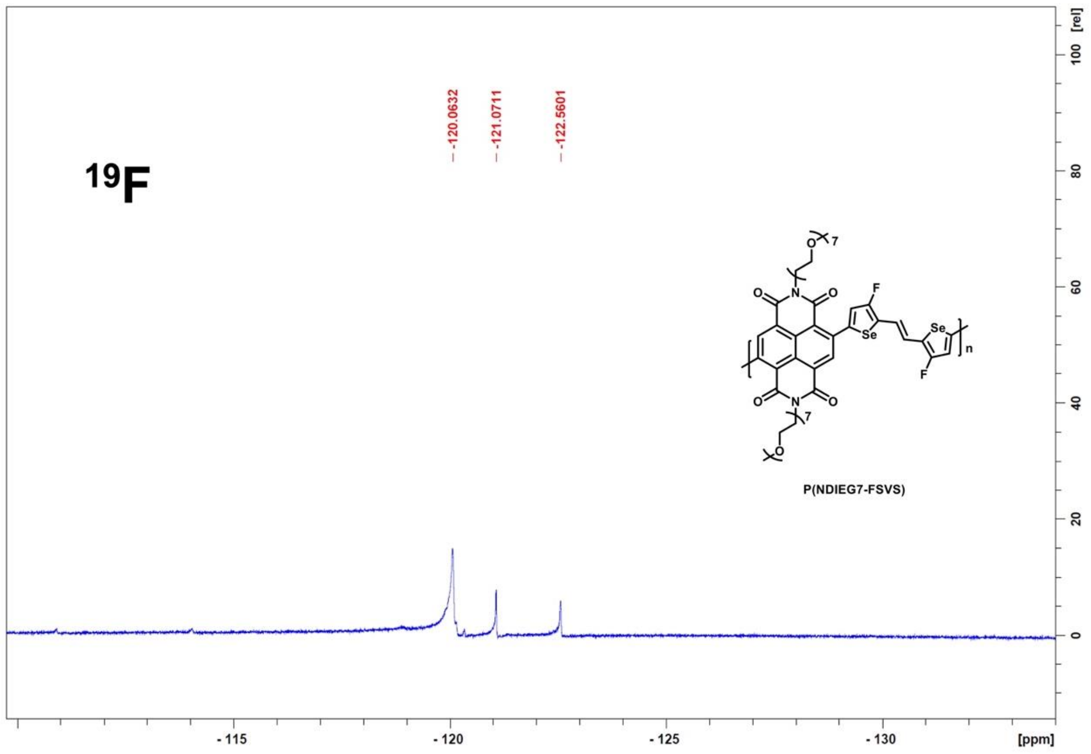

2. Polymer molecular weights, PDIs, and thermal properties

2.1 SEC measurement:

High Temperature-Size Exclusion Chromatography (HT-SEC): SEC characterization was carried out on an Agilent PL-220 High Temperature GPC instrument, using 1,2-dichlorobenzene (DCB) with 250 ppm BHT additive as the eluent, at 140 °C at a flow rate of 1mL min-1. The system is equipped with differential refractive index (DRI), light scattering (LS) and viscometer (VS) detectors, 2 x PLgelOlexis columns (13 µm, 300 x 7.5 mm) and a PLgelOlexis guard column (13 µm, 7.5 x 50 mm). The system was calibrated using polystyrene standards (Agilent Easivials) with molecular weights in the range of 580 – 6,570,000 g mol-1. Prior to injecting the samples, they were prepared on a known concentration and were heated at 150 °C for 3 hours before filtering using a hot filtration device, PL-SP 260 VS.

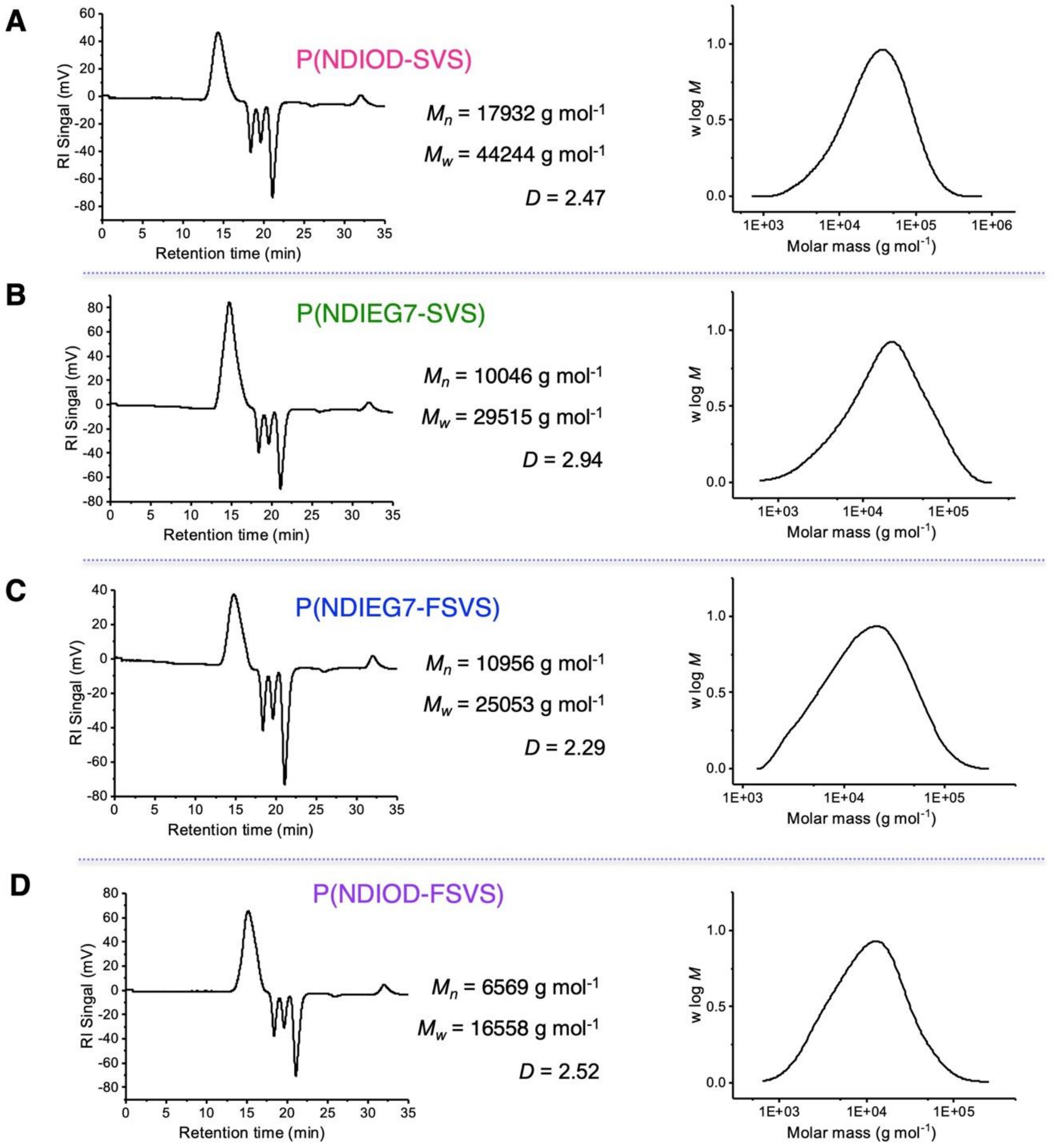

Figure S1: Chromatogram and (C) Molecular Weight Distribution of A A) P(NDIOD-SVS), B) P(NDIEG7-SVS) , C) P(NDIOD-FSVS) , and D) P(NDIEG7-FSVS).

2.2 TGA and DSC measurements:

The TGA results were recorded on a TA Instruments 650 SDT instrument running in a 100 ml min^{-1} nitrogen atmosphere. The samples were loaded into 90 µl alumina pans and run in a temperature profile from room temperature to 1000 °C at a heating rate of 10 °C min^{-1}.

All DSCs were run on a Mettler Toledo DSC 1, equipped with liquid nitrogen cooling in a 50ml min^{-1} nitrogen gas atmosphere. The temperature profile used was from -100 to 300 °C in a heat-cool-heat-cool regime, with a 10 °C min^{-1} ramp rate. Samples were loaded into 40 µl aluminum pans with a pinhole top to allow for gas release during the experiment.

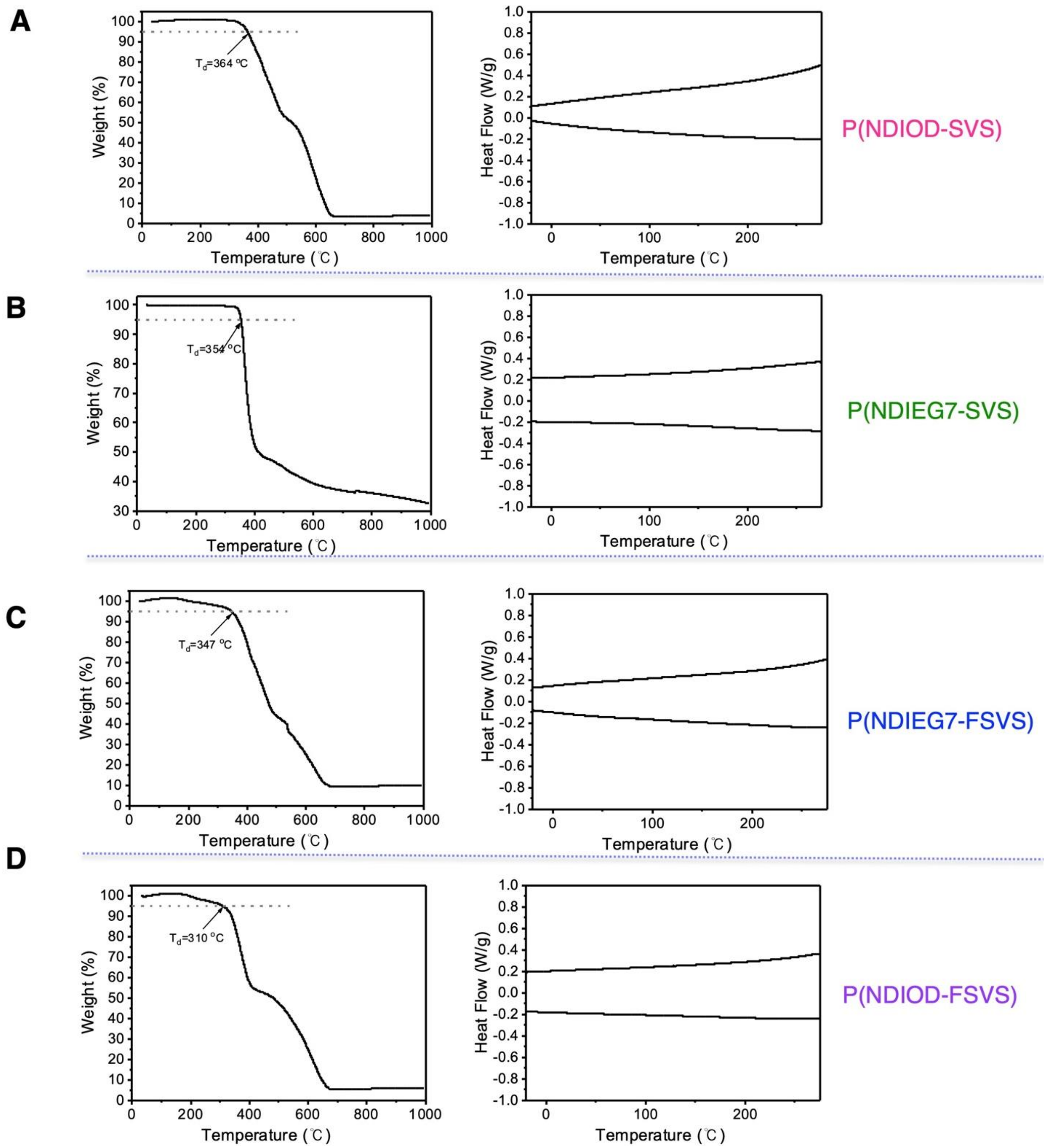

Figure S2: TGA and DSC measurements of A) P(NDIOD-SVS), B) P(NDIEG7-SVS) , C) P(NDIOD-FSVS) , and D) P(NDIEG7-FSVS).

3. In-situ Spectroelectrochemistry Measurements:

The onsets for all polymers are marked and summarized in Table S1 together with the calculated LUMO values. One can see that the onset determination of both methods gives very comparable results.

polymer	E_{onset} [V]		E_{LUMO} [eV]		neutral N	first reduction $R^{\bullet-}$	second reduction D^{2-}
	from CV [V]	from UV-vis absorption spectra [V]	from CV [eV]	from UV- vis absorption spectra [eV]	λ_{max} [nm]	λ_{max} [nm]	λ_{max} [nm]
**P(NDIOD-SVS) 1st Cycle**	-1.03	-1.05	-4.07	-4.05	405, 743	412, 539, 783	438, 599, 938
**P(NDIEG7-SVS) 1st Cycle**	-0.71	-0.69	-4.39	-4.41	418, 786	415, 516, 743, 776	418, 796
**P(NDIOD-FSVS) 1st Cycle**	-0.86	-0.88	-4.24	-4.22	405, 743	402, 539, 770	432, 593, 942
**P(NDIEG7-FSVS) 1st Cycle**	-0.47	-0.48	-4.63	-4.62	415, 743	418, 509, 767	415, 780

Table S1: Onset potentials vs. Fc/Fc^+ with calculated LUMO values and characteristic bands for all polymers.

UV-vis Spectra	Peak Evolution
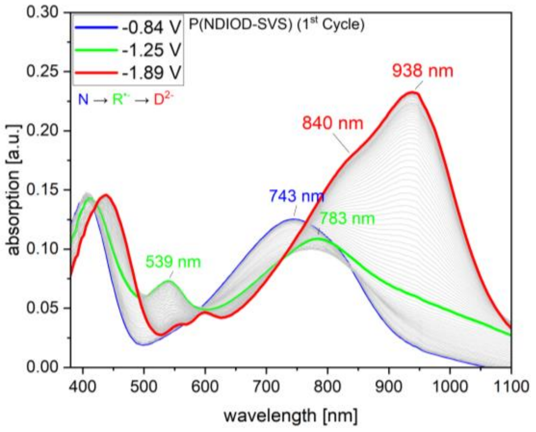	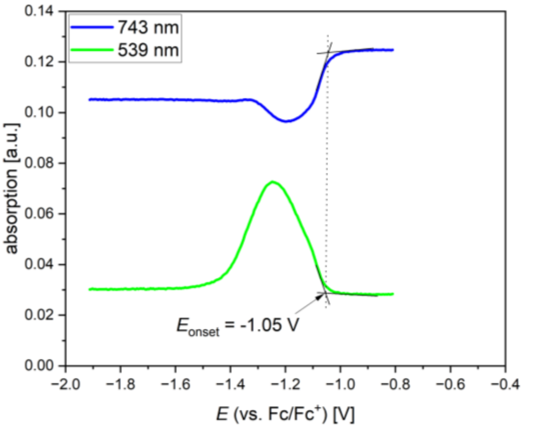
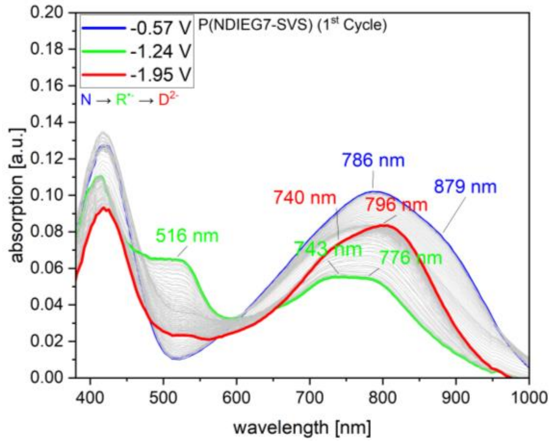	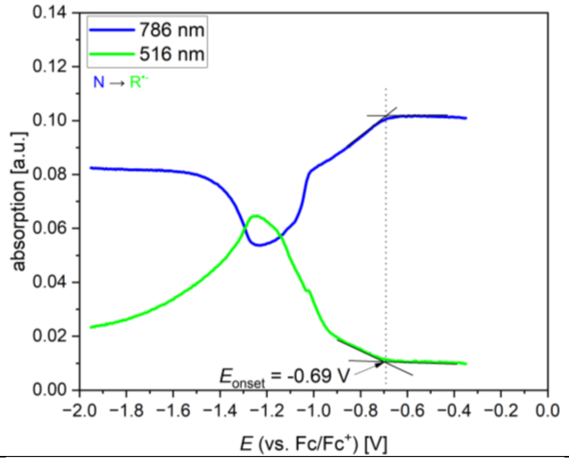
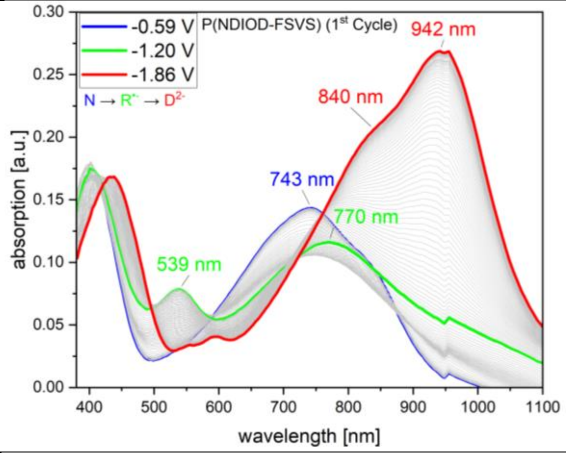	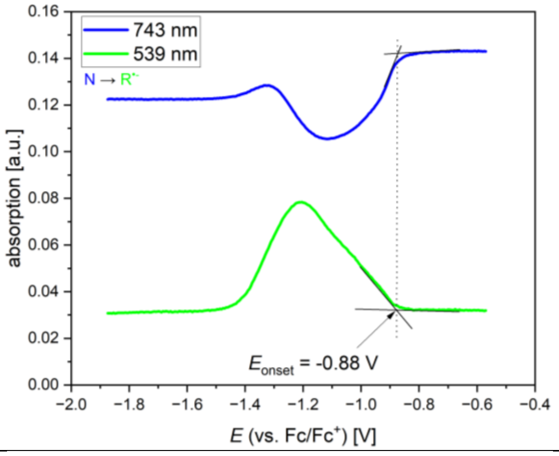
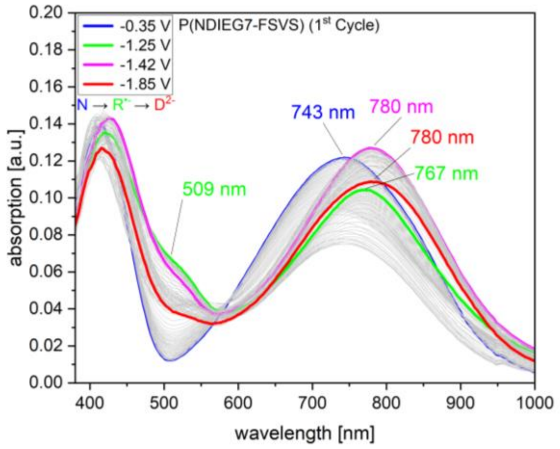	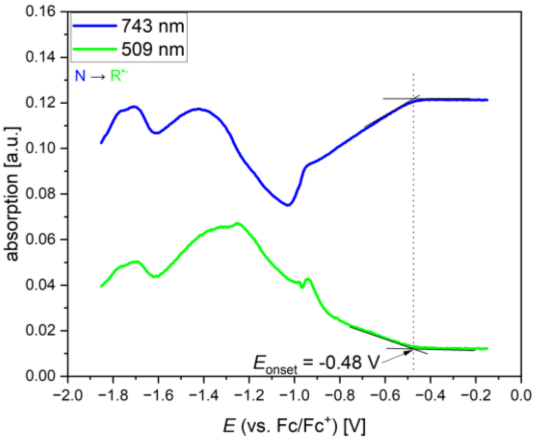

Figure S3: UV-vis spectra and the evolution of the absorption intensity of the neutral and first reduced state during reduction for the spectral onset determination of the polymers.

4. GFN2-xTB and DFT calculations

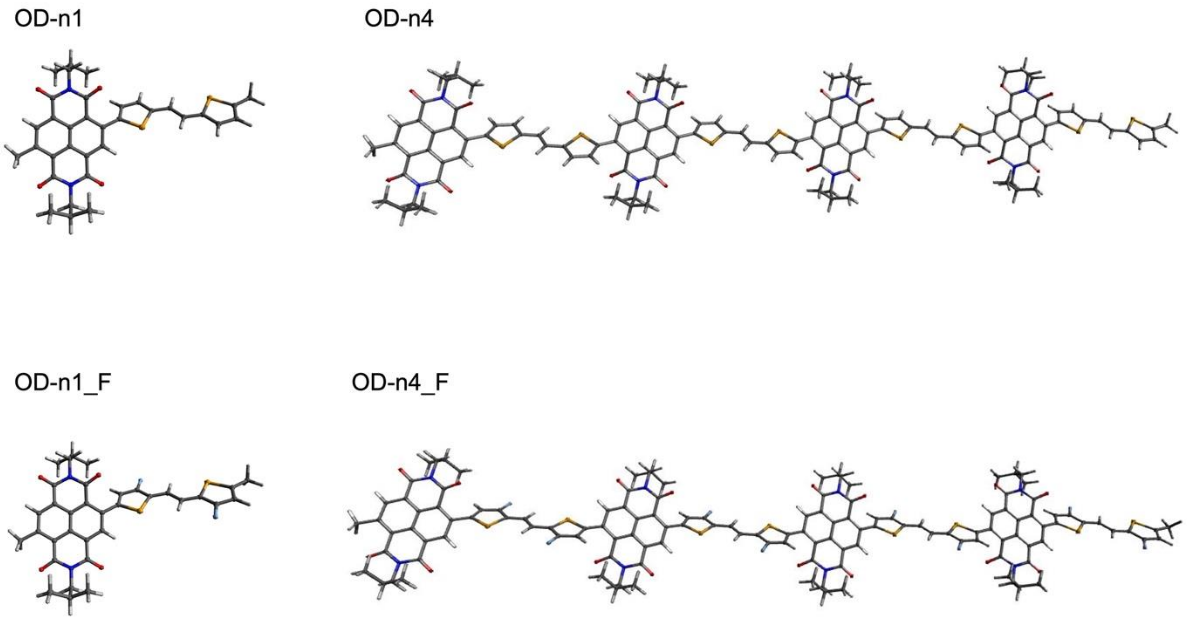

Figure S4: DFT-optimized structures for monomer (n1) and tetramer (n4) of P(NDIOD) species without and with fluorine atoms.

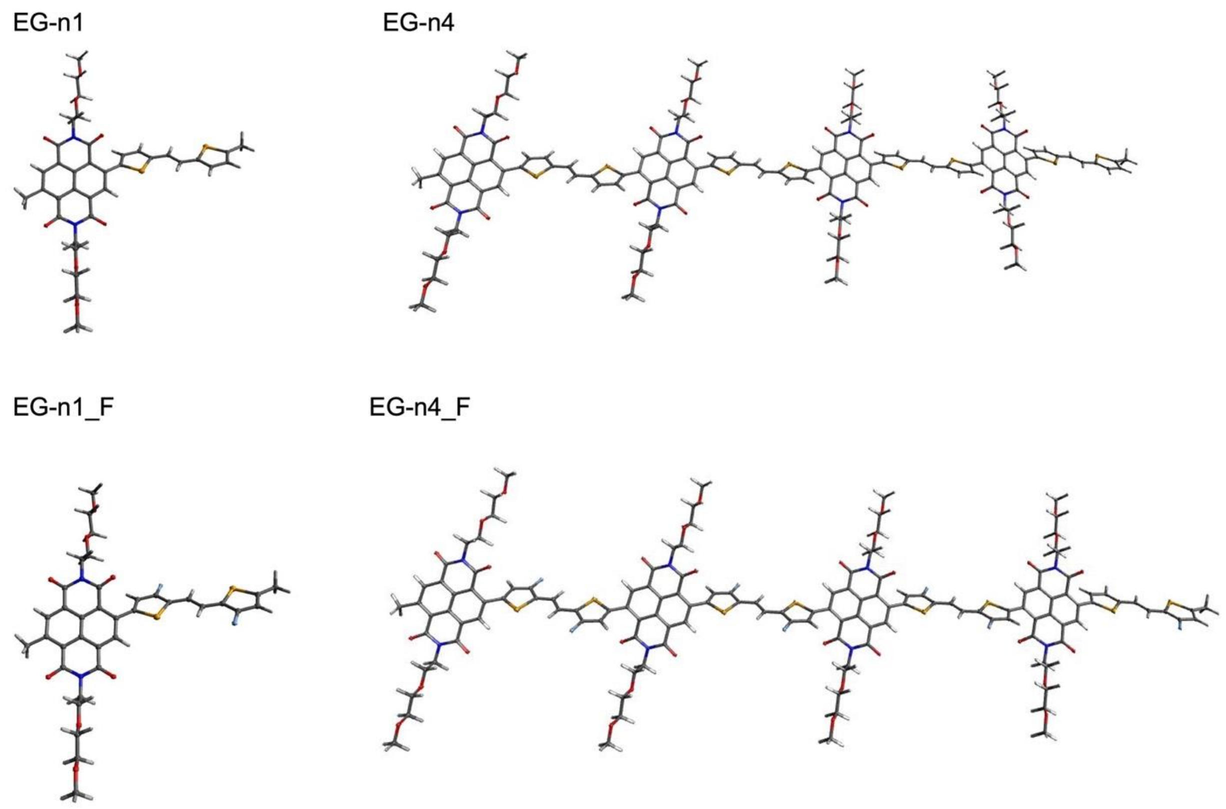

Figure S5: DFT-optimized structures for monomer (n1) and tetramer (n4) of P(NDIEG7) species without and with fluorine atoms.

**P(NDIOD)**	GFN2-xTB	B3LYP-D3BJ (ZORA)
n1 (SVS)	38.8	45.4
n1 (FSVS)	41.2	45.7
n4 (SVS)	< 41.0 >	< 42.1 >
n4 (FSVS)	< 41.2 >	< 40.2 >

**P(NDIEG7)**	GFN2-xTB	B3LYP-D3BJ (ZORA)
n1 (SVS)	37.7	43.9
n1 (FSVS)	39.7	45.7
n4 (SVS)	< 41.0 >	< 42.0 >
n4 (FSVS)	< 43.0 >	< 42.6 >

Figure S6: Semiempirical GFN2-xTB and DFT (B3LYP/ZORA-def2-TZVO) computed torsional angles between the NDI plane and the selenophene ring.

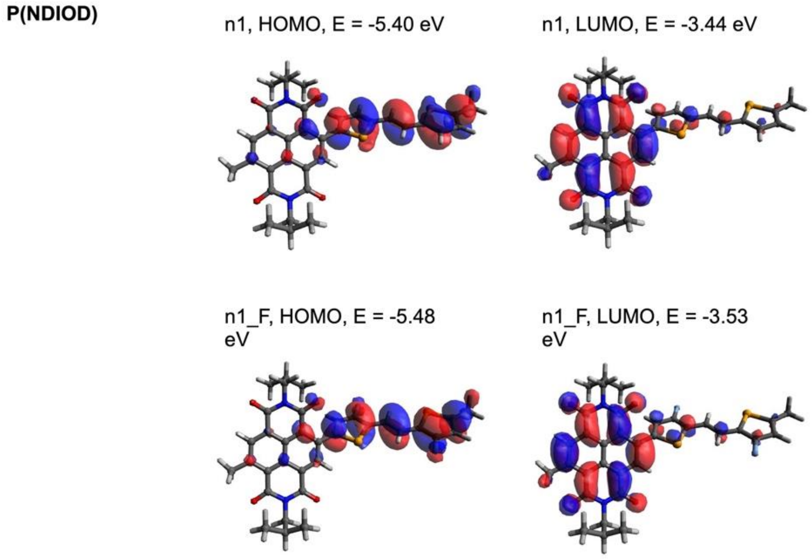

	n1	n1_F	n4	n4_F
HOMO	-5.40	-5.48	-5.29	-5.3
LUMO	-3.44	-3.53	-3.63	-3.74

Figure S7: DFT HOMO and LUMO energies for the monomer and tetramer units of P(NDIOD-SVS and -FSVS).

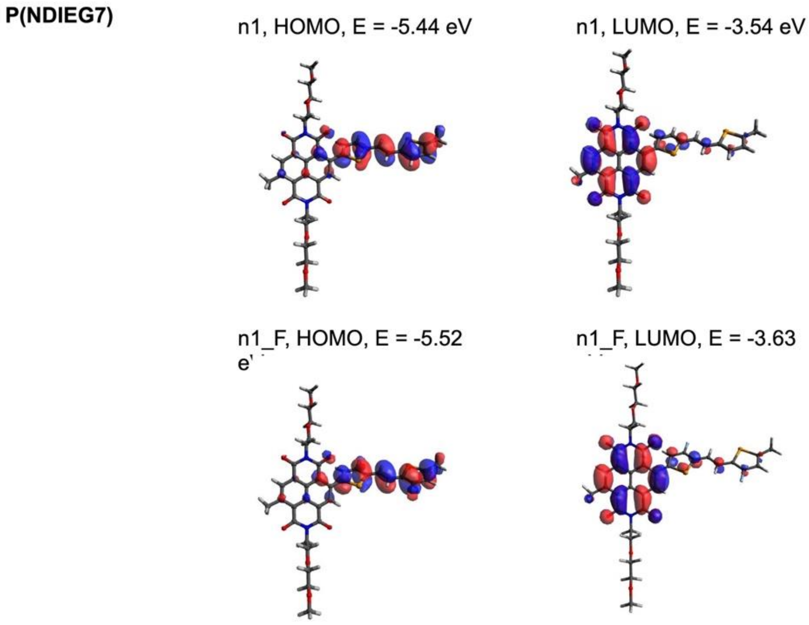

	n1	n1_F	n4	n4_F
HOMO	-5.44	-5.52	-5.24	-5.35
LUMO	-3.54	-3.63	-3.71	-3.82

Figure S8: DFT HOMO and LUMO energies for the monomer and tetramer units of P(NDIEG7-SVS and -FSVS).

5. Impedance measurements for P(NDIEG7-FSVS)

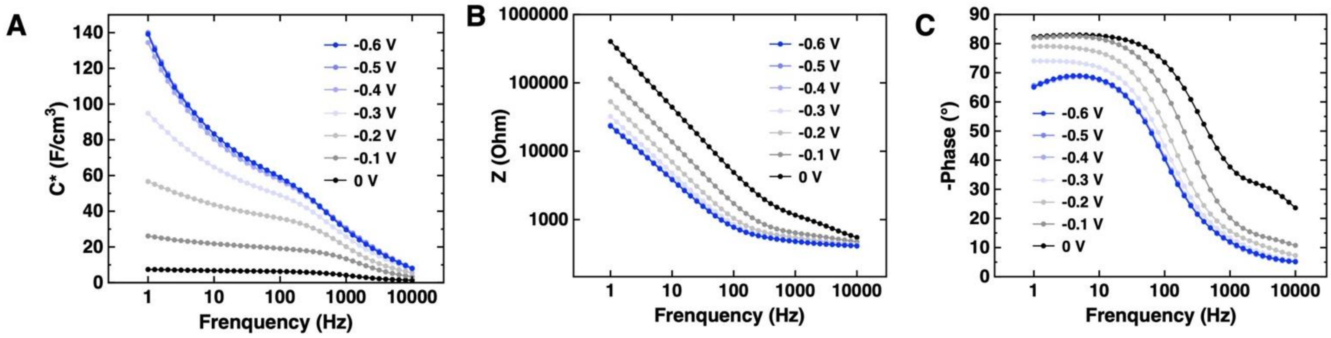

Figure S9: Impedance measurements for P(NDIEG7-FSVS): (A) capacitance, (B) total impedance, and (C) phase angle under different DC bias. Note that since the DC bias is applied to the polymer, the sign of the voltage is thus negative.

6. AFM images of P(NDIOD-SVS) and P(NDIOD-FSVS)

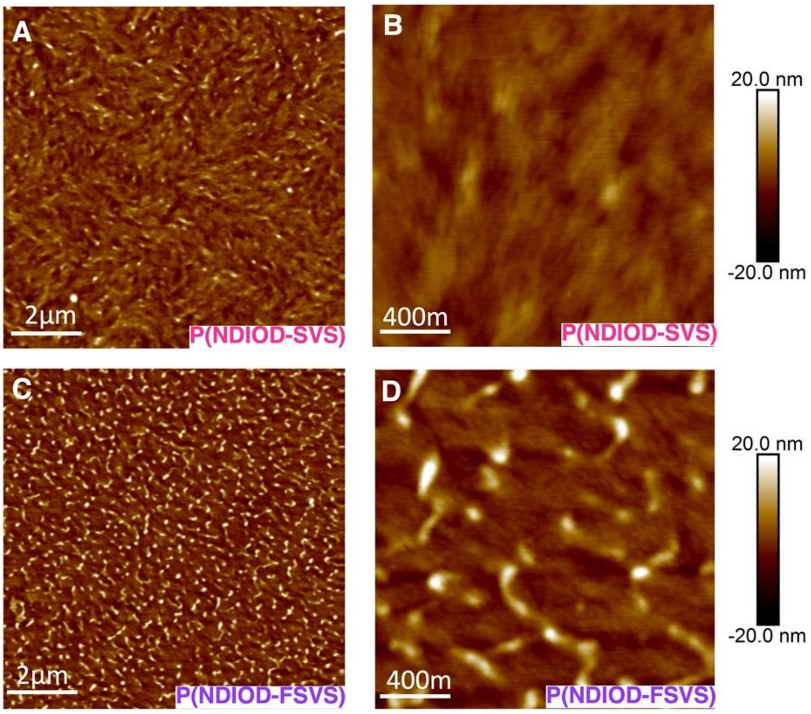

Figure S10. Tapping-mode AFM images of of A), B) P(NDIOD-SVS), and C), D) P(NDIOD-FSVS).

7. Low-temperature OFET transfer characteristics of devices employing P(NDIOD-FSVS)

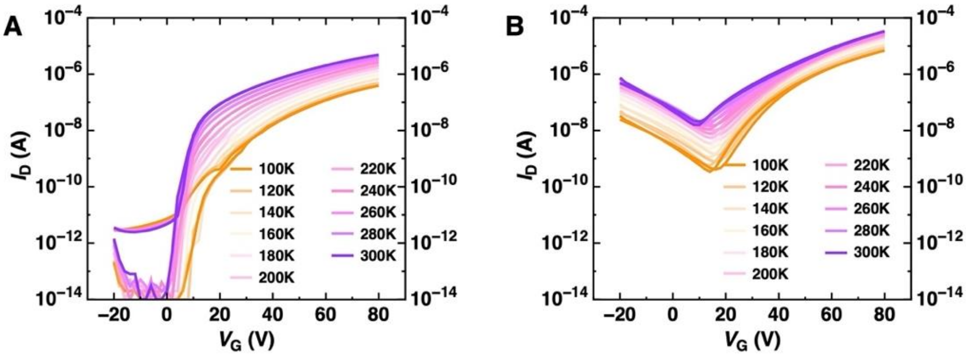

Figure S11: OFET transfer characteristics measured on OFET devices of P(NDIOD-FSVS) at low-temperatures: (A) Linear, and (B) Saturated regime.
